# Older birds have better feathers: A longitudinal study on the long-distance migratory Sand Martin, *Riparia riparia*

**DOI:** 10.1371/journal.pone.0209737

**Published:** 2019-01-04

**Authors:** Tibor Szép, János Dobránszky, Anders Pape Møller, Gareth Dyke, Ádám Z. Lendvai

**Affiliations:** 1 Institute of Environmental Science, University of Nyíregyháza, Nyíregyháza, Hungary; 2 Department of Materials Science and Engineering, Budapest University of Technology and Economics, Budapest, Hungary; 3 Laboratoire Ecologie, Systematique et Evolution, UMR 8079 CNRS-Université Paris-Sud XI-AgroParisTech, Université Paris-Sud XI, Orsay Cedex, France; 4 Department of Evolutionary Zoology and Human Biology, University of Debrecen, Debrecen, Hungary; 5 Department of Geology, Babes-Bolyai University, Cluj-Napoca, Romania; Fred Hutchinson Cancer Research Center, UNITED STATES

## Abstract

Feather quality is of critical importance to long-distance migratory birds. Here, we report a series of analyses of a unique data set encompassing known-age individuals of the long-distance migratory Sand Martin (*Riparia riparia*). Sampling over 17 years along the Tisza River, eastern Hungary, has resulted in the recapture of numerous individuals enabling longitudinal and cross-sectional investigation of the role of adaptation to variable environmental conditions on feather morphology. We show that older individuals tend to possess better quality feathers, measured using bending stiffness, feather length and thickness as proxies. Bending stiffness and feather thickness do not change with individual age, in contrast with increases in feather length and declines in daily feather growth versus age of individual alongside moult duration. Individuals who live to older ages tend to have similar, or higher, feather growth rates and better feather quality than individuals captured at younger ages. Thus, on the basis of strong selection against individuals with slow feather growth, as seen in other species of swallows and martins, which causes a delay in moult completion, the results of this analysis highlight the potential cost of producing better quality feathers when this depends on moult duration. Feather length also does change during the lifetime of the individual and thus enabled us to further investigate influence of individual and environmental conditions during the moult. The results of this analysis provide important insights on the adaptive significance of these traits, and the potential use of physical characteristics in unravelling the reasons why long distance migratory bird populations are in global decline.

## Introduction

Feathers are unique and complex biological integumentary structures that are incrementally grown daily and periodically replaced by birds [1]. Feather condition is critical to the success and survivorship of birds; without good quality, functional feathers, birds cannot fly effectively, forage, or attract a mate [2,3]. The moulting of feathers is at the same time unavoidable because old feathers become abraded and worn due to mechanical friction, exposure to sunshine, ectoparasites, bacteria and other environmental factors [4].

Moult is energetically, but also locomotory demanding [5]. Therefore, the maintenance of adequate feather quality and quantity is traded against other activities throughout a bird’s lifecycle. This is especially important for migratory birds. Feather quality has been shown to determine sedentary time for moult and migration [6, 7], especially in long-distance migratory bird species [8]. Feather durability also depends to a large extent on quality [9], and high quality feathers reduce the probability of moult initiation during migration [10]. Gaps in the wings or tail hamper flight performance and increase metabolism over long periods [11]. Feather damage also increases an individual’s risk of death due to predation [12] and risk of collision.

Feather quality can be measured in a range of different ways (e.g., feather mass, mass relative to the feather length, rachis diameter, bending stiffness, level of melanisation, occurrence of abnormalities [6, 9, 13, 14, 15, 16]). Among these measurements bending stiffness is the variable that reflects most directly the mechanical property of feathers because it transmits aerodynamic forces to the musculoskeletal system during flight, and it is therefore related to feather durability [6, 13]. Bending stiffness is the physical outcome of two mechanical parameters, the Young modulus (the ratio of the tensile stress to strain) and the second moment of area (estimated via the rachis dorsoventral width of a feather). There are only a few detailed investigations of these parameters responsible for bending stiffness [17] in relation to moult [6, 13, 18, 19]. For example, an experimentally increased speed of moult has been shown not to affect Young modulus, but affected the rachis dorsoventral width of moulted feathers, which became less rigid [13]. Studies to compare to the long-distance migratory willow warbler *Phylloscopus trochilus*, which moults twice a year versus the shorter-distance migratory chiffchaff *P*. *collybita* which moults just once a year [6, 19] have shown that the former can increase feather stiffness by increasing rachis dorsoventral thickness via low-quality keratin, but this results in an elastic loss in Young’s modulus. These birds then pay for this trade-off by experiencing higher mechanical fatigue rates which together lead to lower feather durability [18].

Timing of moult may also affect feather quality. Limited moult duration impairs feather quality [13, 14, 20, 21]. In general, since feather quality is negatively affected by faster feather growth [22, 23], only birds in prime condition are able to moult fast without compromising their feather quality [14].

Fully grown feathers are inert structures, therefore, with characteristics that reflect the conditions experienced by birds during moult. This can provide especially important insights for long-distance migrants that moult in distant, often remote areas. Feather characteristics can be useful tools for investigating evolutionary and ecologically relevant parameters as they act seasonally and trans-seasonally in moulting areas and influence the fitness of birds [24, 25, 26]. This is important because many European and North American populations of long-distance migratory birds have declined dramatically over the last two-to-three decades, in contrast to resident and short-distance migratory species [27]. However, while detailed information is available for the breeding season, data relevant to the non-breeding period, which largely overlaps with moulting in migratory species, is lacking [28].

Few studies have investigated how feather morphology changes over time [23, 24, 29, 30, 31], although detailed cross-sectional approaches have sometimes been adopted [6, 13, 32]. A good deal of information is available on how internal and external factors influence feather morphology, but relatively little is known about how physical parameters change with the age of individuals [23, 33], and how plasticity allows free-living long-distance migratory animals to respond to the different environments they encounter [24].

We examined a large and unique data set of feather traits from known-age individuals of the short-lived, long-distance migratory Sand Martin (*Riparia riparia*). Intense sampling over 17 consecutive years has captured many known age individuals repeatedly so our data enables both longitudinal (e.g., the same individuals producing different feathers as they grow older) and cross-sectional (e.g., younger vs older individuals) investigation into the role of plasticity and natural selection on feather morphology. We hypothesise that improved feather quality, measured as feather size, mass, rachis width and bending stiffness all enhance survivorship. We therefore predict that older birds will have better quality feathers [13] compared to their younger counterparts, or even the same individuals when younger, resulting in differences in feather quality when comparing analyses of longitudinal and cross-sectional data.

## Material and methods

### Study species

The European Sand Martin is a small (*ca*. 14 g) socially monogamous long distance migratory bird which often breeds in large colonies [34]. This species over-winters and undergoes complete moult in Sub-Saharan Africa [35]; one of the longest (135 days) periods away from the breeding grounds seen in European passerines. Our study population breeds along the banks of the Tisza River in eastern Hungary, uses different African wintering areas [36] and exhibits large annual fluctuations in population size and survivorship [37]. We have had permits from the relevant nature conservation authorities since 1995, especially for collecting feather samples (one pair of second outermost tail feathers). All field work was evaluated by a committee on the base of conservation and ethical considerations. Field permits were granted, at various time depending on Hungarian national legislation, by the Hortobágy National Park (1995–2004), the Upper-Tisza Inspectorate for Environmental Protection, Natural Protection and Water Management (2005–2008), and the General Directorate of Environmental Protection, Natural Protection and Water Management (2009–2020).

### Field data and feather collection

Birds were captured at breeding colonies in mist nets along the Tisza River [38]. A total of 129,179 individuals were captured by TSz and teams between 1995 and 2011, of which 12,225 were subsequently recaptured. Field teams plucked the second outermost tail feather (T5) from both sides of selected adult individuals that had moulted in Africa, and for which age and nest were known. Our resultant sample comprises 12,260 pairs of feathers harvested between 1995 and 2011, and includes those collected in consecutive years from the same individuals. The analyses presented here are therefore based on the subset of feathers from 148 birds that met the following criteria. The birds were of known age (*i*.*e*., first ringed as nestlings or juveniles), and with tail feathers that were collected in two different years between 1995 and 2011. Feathers from 31 additional individuals aged between four and seven, or older, were also considered, even though specimens from previous years were unavailable (179 birds in total). Sex and biometric data (*i*.*e*., wing length, tail length, body mass, keel, and tarsus length) were also measured.

The return rate of individuals whose feathers had been removed was compared with a control group that was captured simultaneously, but that did not have their feathers removed. Feather sampling had no significant effect on return rate between 1995 and 2011 in either sex; the return rate of males was 10.5% (244/2,329) and 9.7% (491/5,070) in sampled and control birds, respectively (χ^2^ = 1.119, df = 1, P = 0.296), while in females the return rate was 9.0% (215/2,385), and 8.7% (627/7,202) (χ^2^ = 0.213, df = 1, P = 0.646), respectively.

Date of capture (date of first capture in the given year) in our research is an important variable which has two biological interpretations. In the first place, because we captured individuals at the colonies at a constant rate during the entire breeding season, the date of first capture in the given year reflects the arrival time of the individuals. From this point of view, the date of capture (i.e. arrival date) may reflect individual quality [15]. Secondly, the date of capture might also affect the condition of the collected feathers, because feathers collected later in the breeding season could be more worn [39] than feathers collected just after the arrival back to the colony.

### Feather measurements and analyses

We measured the length, mass, degree of wear, rachis width, growth bar width, and bending stiffness of each T5 feather. To measure length, we scanned the ventral side (using a Canon CanoScan D660U, 300 dpi) and measured the full length of the rachis to the nearest 0.01 mm using a curve in ImageJ [40]. We also weighed feathers to the nearest 0.01 mg using a digital balance (ADAM, AAA 160DC). Since feather wear can affect other feather parameters (e.g., mass, length), its extent was scored as either intact (0, no visible signs of feather wear) or worn (1, broken ramus at the tip). Intact Sand Martin feathers tended to be collected significantly earlier in the breeding season (day measured as number of day elapsed since 31^th^ of March, day 1: 1^st^ of April); intact feathers, 71.01 days (SD = 20.213, n = 151), compared to worn, 83.65 days (SD = 15.236, n = 176). The mean difference between these data is 12.63 days (SE = 2.006, t = -6.298, df = 275.731, P < 0.001).

Rachis width, the distance between the outside edges of the dorsoventral walls, was measured to the nearest 0.01 mm across the umbilicus superior (*i*.*e*., at the base of the feather vane) using digital Vernier callipers (Powerfix Z11155). Growth bar widths were measured from digital photos taken with a Canon 7D camera in a dark room with shallow angle lighting [41]; we measured the width and position of all visible growth bars from the base of the vane to the feather tip [22] using ImageJ [40]. The width of a growth bar is the distance between the proximal edges of two adjacent dark bands.

Results from a random subset of our complete data show that measurements of feather length, mass, and rachis width are highly repeatable [42] (*i*.*e*., feather length: R = 0.997, SE = 0.001, F_132,133_ = 802.604, P < 0.001; feather mass: R = 0.996, SE = 0.001, F_39,40_ = 451.396, P < 0.001; rachis width: R = 0.923, SE = 0.024, F_39,40_ = 24.807, P < 0.001). Of the parameters we measured on the studied individuals in the field (unusual T5 feathers and T5 feathers with outlier values excluded), T5 length significantly correlated with wing length (R = 0.450, N = 81, P < 0.001) and tail length (R = 0.51, N = 79, P < 0.001), T5 mass significantly correlated with wing length (R = 0.318, N = 81, P = 0.004) and tail length (R = 0.374, N = 79, P < 0.001), and T5 rachis width significantly correlated with body mass (R = 0.27, N = 81, P < 0.014) and keel length (R = 0.241, N = 77, P = 0.034).

As each single feather growth bar is produced each day [22], we used mean growth bar width (GBW) as a surrogate for average daily growth. Thus, using all visible individual growth bars, we calculated mean GBW for each feather. Repeatability of this measure was significant (R = 0.645, SE = 0.094, F_39,40_ = 4.815, P < 0.001) for a randomly selected subset of feathers.

We measured the dorsoventral bending stiffness of feathers using a Zwick 005 testing machine [6, 43]. In total, 327 feathers were cantilevered 1 cm from the base (calamus) and loaded at 2 mm/min, 38 mm from the rachis base (two-thirds mean sampled feather length) until 6 mm displacement [43]. In total, *ca*. 3,900 data points were recorded for each feather, which allowed us to estimate bending stiffness as the slope from the initial, linear phase of a load-displacement curve. These bending stiffness measurements were highly repeatable and significant (R = 0.993, SE = 0.001, F_287,288_ = 267.4, P < 0.001) across a randomly selected subset of feathers.

We controlled for length, mass, and rachis width during bending stiffness analyses because these properties influence stiffness (*i*.*e*., rachis width and mass using the second moment of area and structure of the rachis). Length, mass, and rachis width show very strong and significant positive correlations (*i*.*e*., length *vs*. mass: r = 0.750, P < 0.001; length vs. rachis width: r = 0.319, P < 0.001; mass vs. rachis width: r = 0.547, P < 0.001; N = 314, Pearson correlations). We then applied principal component analysis (PCA) to feather length, mass, and rachis width applying ‘varimax’ rotation to extract statistically independent artificial variables and to avoid collinearity during modelling [44]. We extracted the first two components (eigenvalues: PC_1_ = 1.448 and PC_2_ = 0.837) that explain 93.2% of the total variance (70% and 23%, respectively). Of these, PC_1_ was strongly positively-related to feather length (r = 0.958) and feather mass (r = 0.833), and, to a lesser extent, rachis width (r = 0.199); therefore, this parameter reflects feather longitudinal size. PC_2_, on the other hand, was strongly positively correlated with rachis width (r = 0.972) and less so with feather mass (r = 0.436) and length (r = 0.098); therefore, this parameter is likely related to feather thickness.

We used general linear mixed (GLMM) models in all other analyses with individuals as random factors. Assumptions (i.e., homogeneity of variance and the normal distribution of residuals) as well as model fit were then verified using graphical diagnostic tools [44]; models were selected on the basis of AIC values using the ML method, and parameter estimates were calculated from the final model refitted using REML [44]. We used within-subject centring to separate age effects among- and within-individuals (i.e., cross-sectional *vs*. longitudinal comparisons) [45]. Our initial models contained among- and within-individual age effects, sexes, and years. For models of feather length, mass, and bending stiffness, degree of feather wear and growth bar width were included in initial model structures, parameters were removed in a stepwise manner to find the minimal adequate model, and all analyses were completed in the R statistical environment (version 2.15.2) [46].

## Results

### Feather growth

The mean number of visible growth bars in intact (not worn) feathers was 19.14 (SD = 1.565, range 14–24). The daily growth of the feather, based on measurements of the mean width of all visible individual growth bars width, varied with age (Table 1, model 3). The within-individual age component was significantly negatively-related to daily growth; individuals in their older age have shorter daily growth than they did at younger ages. However, the among-individual age component showed the opposite, a weak and non-significant tendency, older individuals had at least similar or longer daily growth than younger ones (within individual age effect: -0.023 (SE: 0.011) mm/year, F_1,138_ = 4.080, P = 0.045, among-individual age effect: 0.012 (SE: 0.008) mm/year, F_1,173_ = 1.990, P = 0.160; Table 1: model 3; S4 Fig).

**Table 1:** Modelling the size, width, and bending stiffness of Sand Martin feathers.

Response	Model No.	Within- ind. age	Among- ind. age	Sex	Year	Feather wear	GBW	PC_1_	PC_2_	df	AIC
*Daily growth (mean growth bar width*, *GBW)*											
	1	×	×	×	×					21	-131.823
	2	×	×	×						6	-139.188
**Final**	**3**	×(-)*	**×(+)ns**.							**5**	**-141.087**
*PC*_*1*_ *Longitudinal size of the feather*											
	4	×	×	×	×	×	×			23	730.904
**Final**	**5**	×(+)***	×(+)***	×*		×(-)***	×(+)***			**8**	**726.789**
	6	×	×			×	×			7	731.657
	7	×	×	×			×			7	749.101
	8	×	×	×		×				7	740.863
*PC*_*2*_ *Thickness of the feather*											
	9	×	×	×	×		×			22	774.080
	10	×	×	×			×			7	757.610
**Final**	**11**	**×**	×(+)***				×(+)***			**6**	**755.805**
	12	×	×							5	761.171
*Bending stiffness*											
	13	×	×	×	×		×	×	×	24	-47.501
	14	×	×	×			×	×	×	9	-65.771
	15	×	×				×	×	×	8	-67.530
**Final**	**16**	**× ns**.	×(+)*					×(+)***	×(+)***	**7**	**-69.487**
	17	×	×					×		6	28.527
	18	×	×						×	6	67.645

Effects included in the given models are denoted by ‘×’.

For models with the lowest AIC values, we report the sign of the effect (+/-), where it is relevant and significance level of the explanatory parameters as follows (* P < 0.05, ** P < 0.01, *** P < 0.001, ns: P > 0.1).

### Feather size

**Fig 1:**
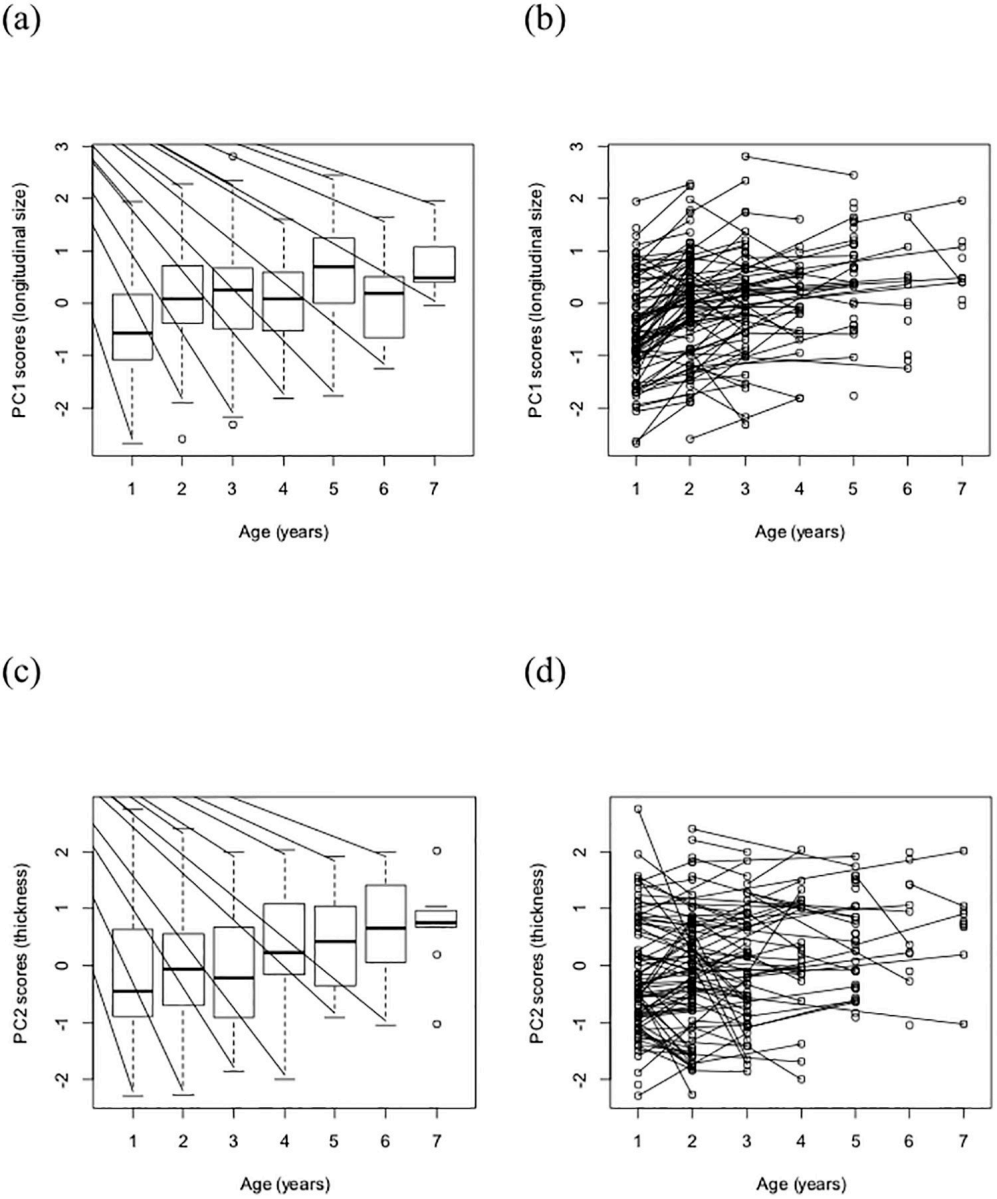
Longitudinal size (PC1) and thickness (PC2) of feathers in different age categories of Sand Martins. (A) Longitudinal size of each age category (*i*.*e*., among-individual age effect: P < 0.001), (B) longitudinal size for the same individual at different ages connected with lines (*i*.*e*., within individual age effect: P < 0.001), (C) thickness for age categories (*i*.*e*., among-individual age effect: P < 0.001), and (D) thickness for the same individual at different ages connected with lines (*i*.*e*., within individual age effect: P = 0.812). Box plots show medians, quartiles, data range (1.5 times the interquartile range) and extreme values.

The longitudinal size of feathers (PC_1_) varied with age, sex, daily feather growth, and degree of feather wear (Fig 1A and 1B, Table 1: model 5). Both within- and among-individual age components were strongly positive (within individual age effect: 0.219 (SE: 0.030), F_1,136_ = 43.720, P < 0.001, among-individual age effect: 0.182 (SE: 0.043), F_1,172_ = 15.751, P < 0.001). The longitudinal size of the feathers was significantly smaller in males (difference between sexes: 0.348 (SE: 0.132), F_1,172_ = 5.311, P = 0.022), positively-related to daily feather growth (GBW: 0.795 (SE: 0.198), F_1,136_ = 16.179, P < 0.001, and was smaller in worn feathers (difference intact and worn feather: -0.387 (SE: 0.078), F_1,136_ = 21.708, P < 0.001). We found the same patterns in feather length and mass (S1 and S2 Figs, S1 Table).

Feather thickness (PC_2_) varied with age; just the among-individual age component was significant (within individual age effect: -0.006 (SE: 0.032), F_1,137_ = 0.057, P = 0.812, among-individual age effect: 0.217 (SE: 0.045), F_1,173_ = 25.119, P < 0.001; Fig 1C and 1D, Table 1: model 11), and positively-related to daily feather growth (GBW) (0.565; SE: 0.208; F_1,137_ = 7.376, P = 0.008). The feathers of older individuals, and individuals with quicker-growing feather, were much thicker (*i*.*e*., had higher PC_2_), but this trait did not change with age of the individuals. We found the same pattern in rachis width (S3 Fig, S1 Table).

### Bending stiffness

**Fig 2:**
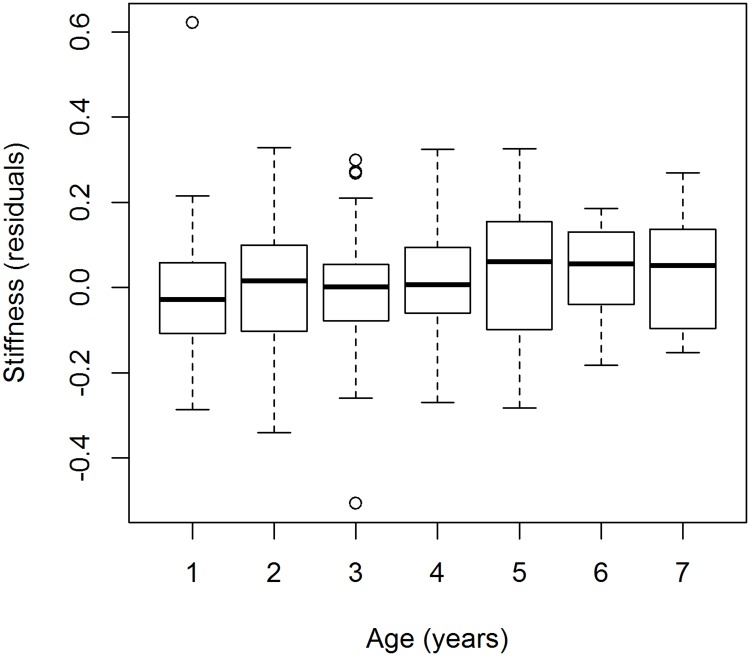
Bending stiffness of feathers of individual Sand Martins in different age categories. Residuals of the best model only consider within and between age, longitudinal size (PC1) and thickness (PC2) for bending stiffness (Model 16, Table 1). Box plots show medians, quartiles, data range (1.5 times the interquartile range) and extreme values.

Bending stiffness of feathers varied among individuals with different age, longitudinal size (PC_1_) and thickness (PC_2_) (Fig 2, Table 1: model 16). Only the among-individual age component was strongly positive for bending stiffness, with older individuals having stiffer feathers than their younger counterparts. However, bending stiffness did not change with the age of individuals when longitudinal size and thickness of feathers were controlled (within-individual age effect: 0.010 (SE: 0.011), F_1,136_ = 0.494, P = 0.484, among-individual age effect: 0.028 (SE: 0.011), F_1,173_ = 6.700, P = 0.011). The size of the feather (PC_1_, PC_2_) was strongly positively related to bending stiffness; longer and thicker feathers had higher bending stiffness (PC_1_: 0.194 (SE: 0.014), F_1,136_ = 234.879, P < 0.001, PC_2_: 0.162 (SE: 0.014), F_1,136_ = 160.844, P < 0.001).

### Spring arrival, age, and feather quality

Feathers were collected when an individual was first captured and this date shows a negative relationship with ageing of the individual and longitudinal size (PC_1_), differing between year of collection (Table 2, model 6). As individuals age, they were caught significantly earlier in the season, but there was no overall difference among individuals of different ages (within individual age effect: -2.667 (SE: 1.122) day/year, F_1,122_ = 10.706, P = 0.001, among individual age effect: -0.067 (SE:0.823) day/year, F_1,173_ = 0.668, P = 0.415).

**Table 2:** Modelling date of Sand Martin capture.

Model no.	Within- ind. age	Among- ind. age	Sex	Year	PC_1_	PC_2_	Bending stiffness	GBW	df	AIC
1	×	×	×	×	×	×	×	×	25	2702.682
2	×	×	×	×	×	×	×		24	2701.178
3	×	×	×	×	×	×			23	2700.338
4	×	×	×	×	×				22	2698.424
5	×	×	×	×					21	2704.302
**6 Final**	×(-)**	**×(ns)**		×***	×(-)**				**21**	**2696.425**
7	×	×			×				6	2733.173
8	×	×		T	×				7	2734.966

Effects included in the given models are denoted by ‘×’.

Year considered as factors denoted by ‘×’, when year considered as numeric variable to model trend denoted with T.

For the models with the lowest AIC values, we report the sign of the effect (+/-), where it is relevant and significance level of the explanatory parameters as follows (* P < 0.05, ** P < 0.01, *** P < 0.001, ns: P > 0.1).

Individuals with longer feathers (PC_1_) were captured earlier (PC_1_: -3.026 (SE: 1.096), F_1,122_ = 7.624, P = 0.007). The date of capture showed a significant difference among years (F_15,122_ = 4.645, P < 0.001), but there was no significant linear trend during the study period (model 6 versus model 8, χ^2^ = 66.541, df = 14, P < 0.001, Likelihood Ratio Test). None of the other measured physical characteristics of feathers had any significant influence on the date of first capture (Table 2).

## Discussion

This study is one of the first to investigate the growth, size, and bending stiffness of feathers naturally moulted by individual birds of known ages across different years. The extent of data collection (over 17 years) reported in this study enabled analysis of both within- and among-individual variation in traits in birds whose age spectrum (from one to seven years old) covered almost the entire range recorded for this species (although the maximum age of individuals in this population is nine years [47] survival rate and telomere length (a molecular marker of biological age) markedly decline after five years of age [48,49]).

Maintaining feather quality may be essential for all birds, but this is of particular importance to our model species, because almost all aspects of their life (foraging, reproduction, and migration) critically depend on flight performance. Sand Martins are exclusively aerial insectivores and long-distance migratory birds; we therefore expect that particularly strong selection operates to keep their feathers in an impeccable state.

We studied tail feathers in order to minimise the effects of sampling on flight performance, but our results may apply to flight feathers as well. Previous studies have shown that tail feathers can be used to predict the growth and moult of the primary feathers in swallows as these moult at similar times [24, 25, 35, 50]. The less-forked tail of the Sand Martin, compared to the deeply-forked tail of other swallow species, including the intensively-studied Barn Swallow (*Hirundo rustica*), may be the result of increased selection to resist lifting forces [51]. Thus, the bending stiffness of tail feathers could provide relevant information on the quality of Sand Martin primary feathers.

Using principal component analysis, we extracted two independent variables describing the length and thickness of feathers, respectively. These feather characteristics varied differently with age. Feather length varied both within- and among-individuals with different ages. This result indicates that feather length is not an individually fixed trait as it changes during the birds’ lifetime. However, even when we statistically controlled for this individual plasticity, the among-individual variation in feather length remained significant, indicating that older individuals in general have longer feathers. Sand Martin individuals in their older ages arrived earlier in the spring to the breeding area, but this varied among years, similar as found in other swallow species [15, 25]. Only feather length showed a relationship with arrival date, while no other measured traits showed such a relationship, similar to previous results [30].

The thickness of a feather showed a different pattern. Feather thickness did not change with individual age, but differed among young and old individuals. Feather thickness therefore does not change during a bird’s lifetime and seems to be an individually fixed trait. This fact in combination with the finding that older individuals had thicker feathers strongly suggests a selective advantage of individuals with thicker feathers. A remarkably similar pattern emerged from the analysis of bending stiffness. Even after controlling for the effects of feather length and thickness (which are expected to influence bending [43]) we found that bending stiffness did not change with individual age, implying limited plasticity in accordance with previous experimental investigations [13], but differed among young and old individuals, with older birds having stiffer feathers. The difference in bending stiffness among individuals could be caused both by rachis cross-sectional geometry, and changing layup, thickness and material properties of keratin laminae but not changes in the Young’s modulus of keratin, because this an intrinsic material property that should be biochemically conserved [17, 52, 53, 54]. Bending stiffness did not change with year of collection, even if it varied in large scale (1–17 years), there is no evidence of degradation of this trait with feather age.

These results suggest that natural selection is favouring individuals with better quality feathers, since only these “superior” quality individuals producing feathers with this kind of structure are able to live for a longer time. Stiffer feathers provide more protection against mechanical wear [18] and will mean lower cost of flight, higher efficiency of foraging and lower threat of predation until the next moult.

Earlier studies [7, 13] regarded feather mass as a proxy of feather quality, and some [30] found that this trait is highly heritable. Our work shows a clear difference in mass among individuals with different ages, although a similar level of within individual difference with age indicates that feather mass refers not only to quality but to longitudinal quantity of feathers (*i*.*e*., longer feathers are heavier) and that this varies with individual age. Our work suggests that bending stiffness reflect better the among-individual variation and that dorsoventral rachis diameter may be an easily measurable proxy for bending stiffness.

Feather quality is also related to moult speed, as feathers experimentally forced to grow faster were of reduced quality compared to control feathers [13,14]. The speed of moult in the Sand Martin, as in several other swallow species that frequently fly long distances, depends predominantly on feather growth rate; these birds have less opportunities to vary their moult intensity because of the high aerodynamic costs of larger wing feather gaps [55]. This may explain why this species comprises one of the slowest moulting in passerines [35].

We found that speed of feather growth declined with individual age, supporting previous findings [29]. This result also reveals that individuals captured at older ages (i.e., those able to reach old ages, over four years in this population [47,48]) are characterised by similar, or probably higher speeds of feather growth as well as higher quality feathers than individuals captured at younger ages (likely not able to reach old age). At the same time, moult duration also increased with individual age because of corresponding increases in feather length and declines in daily feather growth. We found a weaker level of support for differences in the daily growth rates of feathers between the sexes compared to earlier works [24,25]; some researchers have reported faster feather growth in female Barn Swallows, a species that exhibits much larger sexual dimorphism.

In one previous study [24], a Barn Swallow population was studied in their Italian breeding and African moulting areas, and strong selection was shown against individuals with low feather growth who had both slower and later completed moults. The results of this study suggest a potential cost of producing better quality feathers, if this depends on the duration of the moult. Feather growth has heritable component [33], individuals able to grow feathers more quickly have the chance to complete their moult earlier [24], although the speed of feather growth may have to be traded off against feather quality [13, 23, 29, 32, 56]. Individuals with ‘superior’ hereditary qualities and/or enhanced environmental conditions that precede and coincide with the moult can therefore have proper feather growth rate even in their old ages without negatively influencing feather quality.

## Conclusions

The results of this study demonstrate clear evidence for a strong relationship between feather quality and age. We show that bending stiffness and feather thickness (rachis diameter) does not change with the age of the individuals and that the potential costs of producing high quality feathers might relate to the length of the moult, which increases with individual age. A longer moult might result in higher mortality during moult period and/or the spring migration [24]; thus, only individuals able to quickly grow high quality feathers have the chance to complete their moult early, even in old ages. We also show that feather length increases during an individual’s lifetime. Taken together, our results provide important insights on the evolution of feather structure, their adaptive significance, and the potential use of physical feather characteristics for studying the seasonal and cross-seasonal influences on declining long distance migratory bird species.

## Supporting information

S1 Text SM Modelling feather Text. Modelling directly the feather length, mass and rachis dorsoventral width.
(PDF)

S1 Table Results of model selection for Sand Martin investigated feather length, mass and rachis dorsoventral width. (PDF)

S1 Fig Length of the T5 feather (mm) in Sand Martins of different ages. (a) For each age, and (b) length of feathers of the same individual at different ages connected with lines. Box plots show medians, quartiles, 5- and 95-percentiles and extreme values.
(TIFF)

S2 Fig Mass of the T5 feather (mg) among Sand Martins in different age categories. (PDF)

S3 Fig Rachis dorsoventral width (mm) of T5 the feather of different age categories of Sand Martins. (PDF)

S4 Fig Bending stiffness of T5 the feather of different age categories of Sand Martins. (PDF)

S5 Fig Daily growth of the T5 feather (mm/day) of Sand Martins belonging to different age categories. (PDF)

S6 Fig Date of capture versus different age categories of Sand Martins. (PDF)

S1 File Dataset of individuals and their feathers studied in this paper. (XLS)

S2 File SM dataset description. Description of the SM dataset.
(PDF)

S3 File Code for the analysis presented in this paper. (PDF)

S1 Dataset (PDF)
