# Supplementary material for: Older birds have better feathers: A longitudinal study on the long-distance migratory Sand Martin, Riparia riparia
Source: PLoS One. 2019 Jan 4;14(1):e0209737. doi: 10.1371/journal.pone.0209737 (PMC6319700; doi:10.1371/journal.pone.0209737)
Supplement: S1 Text — Modelling directly the feather length, mass and rachis dorsoventral width. (PDF) [file pone.0209737.s001.pdf]

Supplementary material from "Older birds have better feathers: A longitudinal study on the long-distance migratory Sand Martin, *Riparia riparia*"

Tibor Szép[1*], János Dobránszky[2], Anders Pape Møller[3], Gareth Dyke[4], Ádám Z. Lendvai[4]

(a) Feather length, mass and rachis dorsoventral width

Feather length varies with individual age, it differs between sexes, it is positively related to daily feather growth (GBW) and negatively related to degree of feather wear (Fig. S1, Table S1). Parameter estimates show that both within- and among-individual age components are strongly positively related to length (Fig. S1), indicating that feather length increases with individuals age, and that birds with longer lifespan have longer tail feathers than their short lived counterparts (within-individual age effect: 0.388 (SE: 0.062) mm/year, $F_{1,136} = 30.080$, $P < 0.001$, among-individual age effect: 0.353 (SE: 0.086) mm/year, $F_{1,172} = 15.500$, $P < 0.001$, Model 3, Table S1). Females have longer feathers (difference between male and female: -0.613 (SE: 0.265) mm, $F_{1,172} = 3.980$, $P = 0.048$, Model 3, Table S1) and length of the feather is positively related to the daily feather growth (1.928 (SE: 0.406), $F_{1,136} = 22.590$, $P < 0.001$, Model 3, Table S1). The degree of feather wear was negatively correlated with feather length, as expected, since worn feathers were shorter (difference between worn and intact: -0.669 (SE: 0.159) mm, $F_{1,136} = 14.480$, $P = 0.001$, Model 1, Table S1).

The mass of feathers varied with age of the individuals, differed between sexes, was related to the degree of feather wear and positively related to daily feather growth (Fig. S2, Table S1). Both within- and among-individual age components were strongly positively related to feather mass (within-individual age effect: 0.106 (SE: 0.015) mg/year, $F_{1,136} = 39.820$, $P < 0.001$, among-individual age effect: 0.140 (SE: 0.021) mg/year, $F_{1,172} = 39.810$, $P < 0.001$, Model 8, Table S1). This result implies that individuals grow heavier feathers as they become older, and birds that lived longer had heavier feathers. Males had lighter feathers than females (difference between the sexes: -0.185 (SE: 0.066) mg, $F_{1,172} = 5.890$, $P = 0.016$, Model 8, Table S1). The mass of the feather was positively related to the daily feather growth (0.382 (SE: 0.100), $F_{1,136} = 14.730$, $P < 0.001$, Model 8, Table S1) and the degree of feather

wear was negatively related to the mass of feathers, with worn feathers being lighter than intact ones (difference between worn and intact: -0.196 (SE: 0.039) mg, $F_{1,136} = 22.160$, $P < 0.001$, Model 8, Table S1).

Rachis dorsoventral width varied with age and with daily feather growth (Fig. S3, Table S1), individuals with longer lifespan had thicker rachises than others with shorter lifespan, while there was no sign of within-individual change in rachis dorsoventral width with age (within individual age effect: 0.001 (SE=0.001) mm/year, $F_{1,137} = 0.510$, $P = 0.478$, among individual age effect: 0.007 (SE=0.001) mm/year, $F_{1,173} = 31.060$, $P < 0.001$, Model 14, Table S1). Rachis dorsoventral width positively related to the daily feather growth (0.023 (SE=0.006), $F_{1,137} = 13.130$, $P < 0.001$, Model 14, Table S1). This result implies that in contrast with feather length and mass, rachis width does not change as birds become older, but birds that had thicker feather rachises lived longer than those who had narrower rachises, and individuals with higher daily feather growth were able to produce thicker rachises.
