# Supplementary material for: Older birds have better feathers: A longitudinal study on the long-distance migratory Sand Martin, Riparia riparia
Source: PLoS One. 2019 Jan 4;14(1):e0209737. doi: 10.1371/journal.pone.0209737 (PMC6319700; doi:10.1371/journal.pone.0209737)
Supplement: S1 Table — (PDF) [file pone.0209737.s002.pdf]

Table S1. Results of model selection for the investigated feather parameters of Sand Martins. Effects included in the given models are denoted by x. Year considered as factors denoted by x, GBW is the daily growth bar width. For the models with the lowest AIC values, we report the sign of the effect (+/-), where it is relevant and significance level of the explanatory parameters as follows (* P <0.05, ** P < 0.01, *** P < 0.001, #: P = 0.054, ns: P > 0.1).

Response	Model no.	Within-ind. age	Among-ind. age	Sex	Year	Feather wear	GBW	df	AIC
Feather length									
	1	x	x	x	x	x	x	23	1175.709
	2	x	x	x	x	x		22	1188.135
final	***3***	***x(+)****	***x(+)****	***x****		***x(-)****	***x(+)****	***8***	***1171.759***
	4	x	x			x	x	7	1175.130
	5	x	x	x			x	7	1187.284
Feather mass									
	6	x	x	x	x	x	x	23	301.981
	7	x	x	x	x	x		22	311.506
final	***8***	***x(+)****	***x(+)****	***x****		***x(-)****	***x(+)****	***8***	***291.841***
	9	x	x			x	x	7	297.736
	10	x	x	x			x	7	314.048
Rachis diameter									
	11	x	x	x	x		x	22	-1437.615
	12	x	x	x	x			21	-1425.994
	13	x	x	x			x	7	-1455.431
final	***14***	***x ns.***	***x(+)****				***x(+)****	***6***	***-1456.745***
	15	x	x					5	-1445.749
