# Supplementary figures and images for: Older birds have better feathers: A longitudinal study on the long-distance migratory Sand Martin, Riparia riparia
Source: PLoS One. 2019 Jan 4;14(1):e0209737. doi: 10.1371/journal.pone.0209737 (PMC6319700; doi:10.1371/journal.pone.0209737)

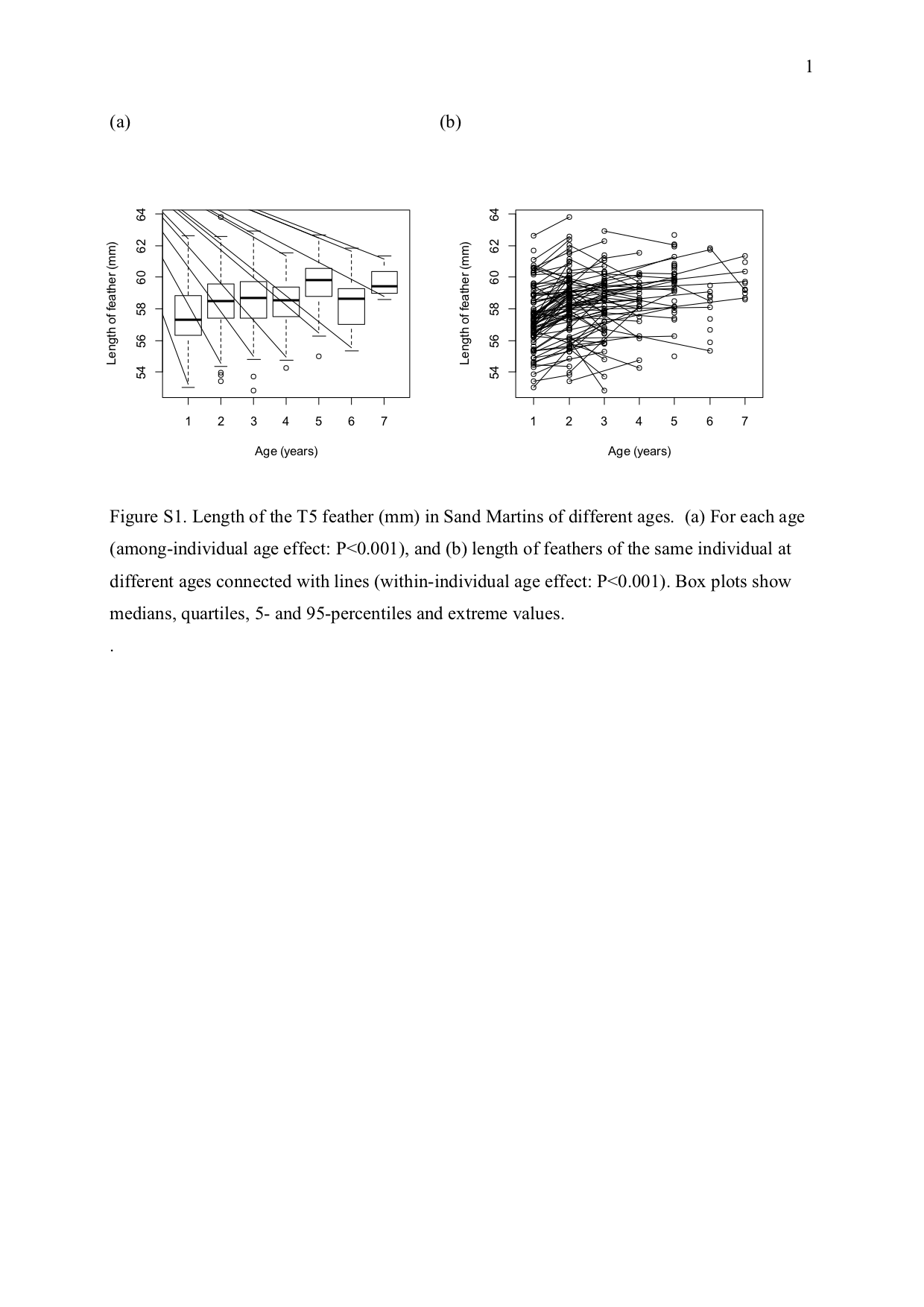

Supplement: S1 Fig — (a) For each age, and (b) length of feathers of the same individual at different ages connected with lines. Box plots show medians, quartiles, 5- and 95-percentiles and extreme values. (TIFF) [file pone.0209737.s003.tiff]
