# Supplementary material for: Older birds have better feathers: A longitudinal study on the long-distance migratory Sand Martin, Riparia riparia
Source: PLoS One. 2019 Jan 4;14(1):e0209737. doi: 10.1371/journal.pone.0209737 (PMC6319700; doi:10.1371/journal.pone.0209737)
Supplement: S2 Fig — (PDF) [file pone.0209737.s004.pdf]

(a) (b)

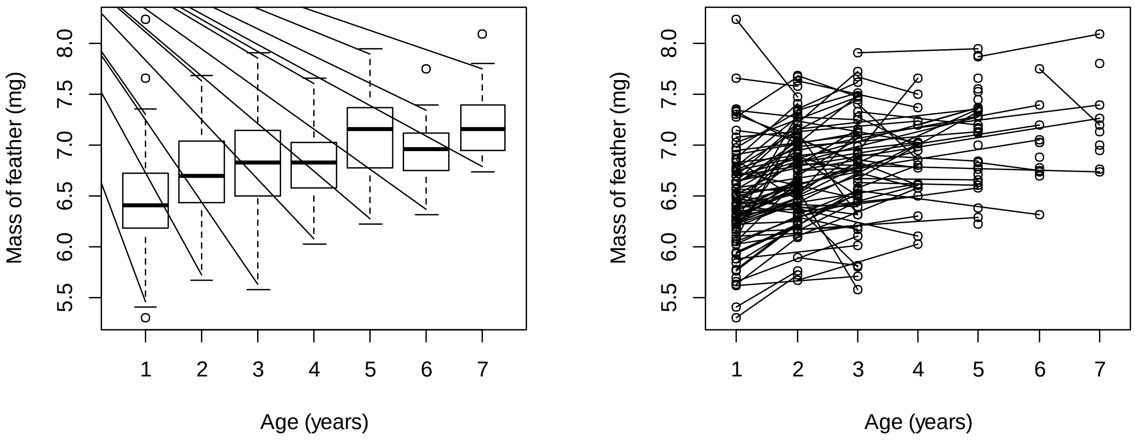

Figure S2. Mass of tail feathers (mg) among Sand Martins belonging to different age categories. (a) For each age category (among-individual age effect: $P<0.001$), (b) mass of feathers (mg) of the same individual at different ages is connected with lines (within-individual age effect: $P<0.001$). Box plots show medians, quartiles, 5- and 95-percentiles and extreme values.
