# Supplementary material for: Older birds have better feathers: A longitudinal study on the long-distance migratory Sand Martin, Riparia riparia
Source: PLoS One. 2019 Jan 4;14(1):e0209737. doi: 10.1371/journal.pone.0209737 (PMC6319700; doi:10.1371/journal.pone.0209737)
Supplement: S4 Fig — (PDF) [file pone.0209737.s006.pdf]

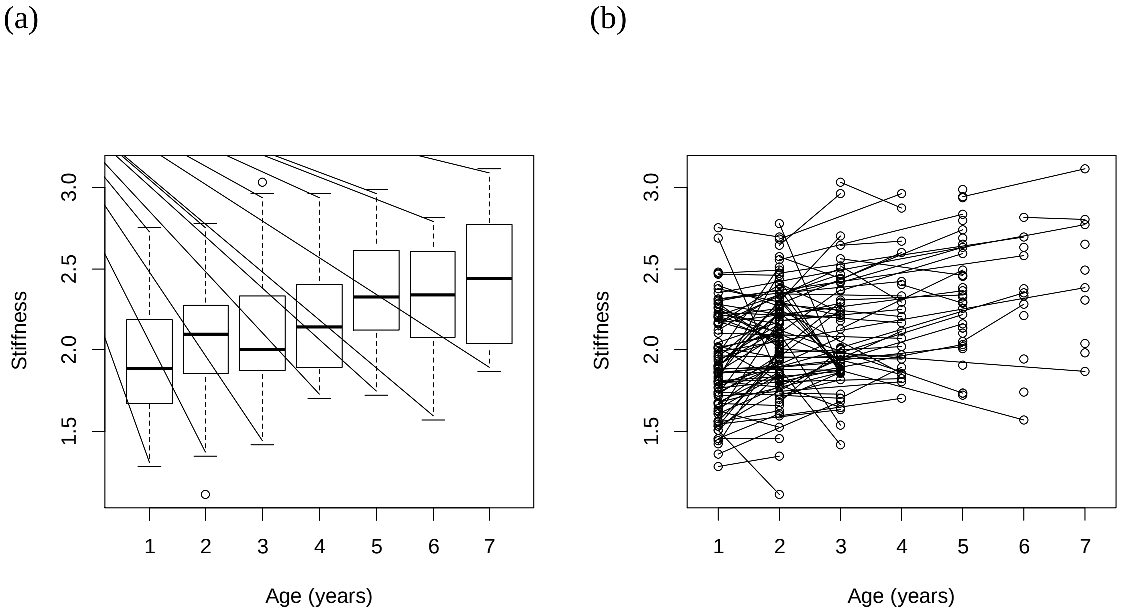

Figure S4. Bending stiffness (slope from the initial, linear phase of a load-displacement curve) of different age categories of Sand Martins. (a) For each age category (among-individual age effect: P=0.011), (b) the same individuals at different ages are connected with lines (within-individual age effect: P=0. 484). Box plots show medians, quartiles, 5- and 95-percentiles and extreme values.
