# Supplementary material for: Older birds have better feathers: A longitudinal study on the long-distance migratory Sand Martin, Riparia riparia
Source: PLoS One. 2019 Jan 4;14(1):e0209737. doi: 10.1371/journal.pone.0209737 (PMC6319700; doi:10.1371/journal.pone.0209737)
Supplement: S5 Fig — (PDF) [file pone.0209737.s007.pdf]

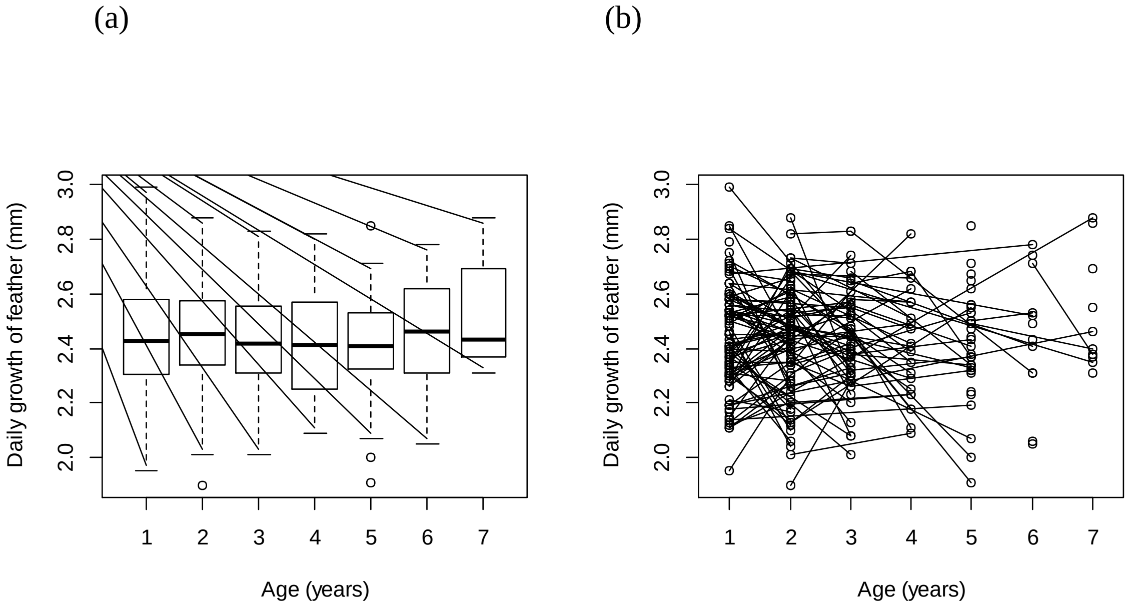

Figure S5. Daily growth of the T5 feather (mm/day) of Sand Martins belonging to different age categories. (a) For each age category (among-individual age effect: P = 0.160), (b) daily growth of feathers of the same individual at different ages are connected with lines (within individual age effect: P = 0.045). Box plots show medians, quartiles, 5- and 95-percentiles and extreme values.
