# Supplementary material for: Older birds have better feathers: A longitudinal study on the long-distance migratory Sand Martin, Riparia riparia
Source: PLoS One. 2019 Jan 4;14(1):e0209737. doi: 10.1371/journal.pone.0209737 (PMC6319700; doi:10.1371/journal.pone.0209737)
Supplement: S6 Fig — (PDF) [file pone.0209737.s008.pdf]

(a) (b)

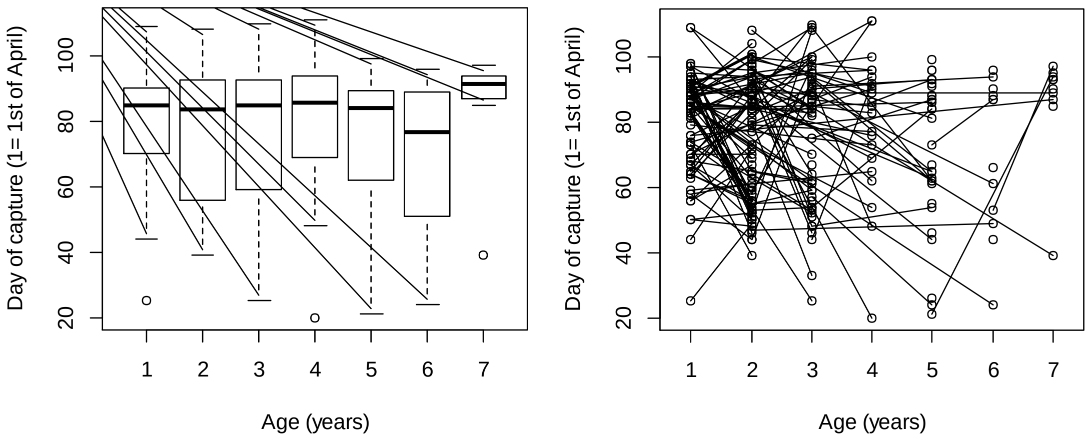

Figure S6. Date of capture vs. different age categories of Sand Martins. (Day 1: April 1st). (a) For each age category (among individual age effect: P = 0.415), (b) date of collection of the same individual at different ages is connected with lines (within individual age effect: P = 0.001). Box plots show medians, quartiles, 5- and 95-percentiles and extreme values. 1 = April 1st Y AXIS.
