# Supplementary material for: Older birds have better feathers: A longitudinal study on the long-distance migratory Sand Martin, Riparia riparia
Source: PLoS One. 2019 Jan 4;14(1):e0209737. doi: 10.1371/journal.pone.0209737 (PMC6319700; doi:10.1371/journal.pone.0209737)
Supplement: S2 File — Description of the SM dataset. (PDF) [file pone.0209737.s010.pdf]

Variables	Description
RING	Ring number
age_ind_b	Age of the individual (1-7 years) one year considered when two samples available for the given individuals from two years
age	Age of the individuals when feather sample was taken (age=1: hatched in the previous year)
sex	Sex, M:male, F: female
year_T5	Year when the T5 feather was taken
bending_T5	Bending stiffness of the T5 tail feathers, measured by the slope of the force-displacement line based on ~3900 measurements/feather (Measured by the same person, JD)
width_T5	Dorsoventral width of the shaft of T5 feathers (mm) where the vane part start, measured by digital caliper (measured by the same person, TSz)
length_T5	Length of the shaft measured on scanned image (mm) (Measured by the same person, EM)
mass_T5	Mass of the T5 feather (mg) (measured by the same person, EM)
weird_cat_T5	Weird category of the measured T5 feather, 0: no evidence, 1: indication of weird condition (measured by the same person, TSz)
ALL1B	Mean length of one bar (mm) (measured by the same person, TSz)
day	day of first caugth, when feather was taken, calculated by day since end of March, 1: 1st of April
RINGER	Code of the ringers who measured biometry in the field
wing_length	Length of the left wing (mm)
mass_g	Mass of the bird (g) at caugth
tarsus	Lenght of the tarsus (mm)
keel	Length of the keel (mm)
tailm	Lenght of the tail (mm), mean of left and right tail (T6) feathers measured in the field
