# Supplementary material for: Older birds have better feathers: A longitudinal study on the long-distance migratory Sand Martin, Riparia riparia
Source: PLoS One. 2019 Jan 4;14(1):e0209737. doi: 10.1371/journal.pone.0209737 (PMC6319700; doi:10.1371/journal.pone.0209737)
Supplement: S3 File — (PDF) [file pone.0209737.s011.pdf]

```
#data
# R 2.15.2 for manuscript
setwd("C:/000/00_r/bend")

library(RODBC)
kapcs = odbcConnectExcel("SM_bending.xls")
benda = sqlFetch(kapcs, "data")
variables = sqlFetch(kapcs, "variables")
odbcCloseAll()

names(benda)[names(benda) %in% "ALL1B"] = "gbar"

#load packages
require(nlme)
require(lme4)
require(lattice)
source("C:/000/00_r/OwnFunctions.r")

#checking and removing outliers!!!
bpl = boxplot(benda$mass)
bpl$out
bpm = boxplot(benda$mass_T5)
bpm$out
bpw = boxplot(benda$width_T5)
bpw$out
# >0.90 outlier for width_T5

bend1 = benda[-which(benda$width_T5 > 0.88 | benda$width_T5 <
0.74),]

bend = bend1[-which(bend1$length_T5 > 64.15 | bend1$length_T5 <
52.68),]

cor.test(~length_T5+mass_T5, data = bend)
cor.test(~length_T5+width_T5, data = bend)
cor.test(~mass_T5+width_T5, data = bend)

# correlation between T5 and field measurement, 1 sample/measurement
per 1 individual, weird feathers and outliers excluded
biometr1 = bend[bend$age_ind_b > 0,]
biometr = biometr1[biometr1$weird_cat_T5 == 0,]

cor.test(~length_T5+wing_length, data = biometr)
cor.test(~length_T5+mass_g, data = biometr)
cor.test(~length_T5+tailm, data = biometr)
cor.test(~length_T5+keel, data = biometr)
cor.test(~length_T5+tarsus, data = biometr)

cor.test(~mass_T5+wing_length, data = biometr)
cor.test(~mass_T5+mass_g, data = biometr)
cor.test(~mass_T5+tailm, data = biometr)
cor.test(~mass_T5+keel, data = biometr)
cor.test(~mass_T5+tarsus, data = biometr)
```

```
cor.test(~width_T5+wing_length, data = biometr)
cor.test(~width_T5+mass_g, data = biometr)
cor.test(~width_T5+tailm, data = biometr)
cor.test(~width_T5+keel, data = biometr)
cor.test(~width_T5+tarsus, data = biometr)

#Approach 2- a: PCA on allometric variables (mass, length, width)
feathervars2 = formula(~length_T5 + width_T5 + mass_T5)
featherPCA <- princomp(feathervars2, data = bend, cor = TRUE)
#featherPCA <- prcomp(feathervars2, data = bend, scale. = TRUE)
#give the same results within rounding error

screeplot(featherPCA)
summary(featherPCA)
biplot(featherPCA)
with(bend, eigen(cor(cbind(length_T5 ,width_T5 , mass_T5))))

#using varimax option like in SPSS
library(psych)
featherPCA.varimax <- principal(subset(bend, select =
attr(terms(feathervars2), "term.lab")),2, rotate = "varimax", scores
= T)
summary(featherPCA.varimax)

#or if we want to use the scores from varimax option
bend$vmx1 <- featherPCA.varimax$scores[,1]
bend$vmx2 <- featherPCA.varimax$scores[,2]

# correlation between PCs and T5 biometry
cor.test(~length_T5+vmx1, data = bend)
cor.test(~mass_T5+vmx1, data = bend)
cor.test(~width_T5+vmx1, data = bend)

cor.test(~length_T5+vmx2, data = bend)
cor.test(~mass_T5+vmx2, data = bend)
cor.test(~width_T5+vmx2, data = bend)

#---------------------PROJECT 2, AGE------------------------------

#-----------1. We analyse bending controlling for PCA variables

#only known age birds, coded with a single number, (e.g. 22 = at
least 2y bird)
age =  droplevels(bend[bend$age<10,])
table(age$RING)

#separating WITHIN and BETWEEN-SUBJECT difference
age$BS_age = ave(age$age, age$RING)
age$WS_age = age$age - age$BS_age


#cor.test(~gbar+length_T5, data = age)
#cor.test(~gbar+mass_T5, data = age)
#cor.test(~gbar+width_T5, data = age)
```

```
#cor.test(~gbar+day, data = age)
age$weird_cat_T5 <- as.factor(age$weird_cat_T5)
#t.test(gbar~weird_cat_T5, alternative='two.sided', conf.level=.95,
#  var.equal=FALSE, data=age)

#t.test(moultday~weird_cat_T5, alternative='two.sided', conf.level=.
95,
#  var.equal=FALSE, data=age)

age_nw =  droplevels(age[age$weird_cat_T5 == 0,])
#cor.test(~moultday+length_T5, data = age_nw)
#cor.test(~moultday+mass_T5, data = age)
#cor.test(~moultday+width_T5, data = age)
#cor.test(~moultday+day, data = age)


save(list = c("age", "bend"), file = "BendingData29.RData")


#----MIXED MODELS

#-----------------

#------- length_T5 general age effect
length_T5.model_age = lme(length_T5~age_ind_b, random = ~1|RING,
data = age, na.action = na.omit, method = "REML")
summary(length_T5.model_age)
anova(length_T5.model_age)

# full model test random year_T5 REML
length_T5.model6n = lme(length_T5~sex+ WS_age + BS_age
+weird_cat_T5, random = ~1|RING, data = age, na.action = na.omit,
method = "REML")
length_T5.model6r = lme(length_T5~sex+ WS_age + BS_age
+weird_cat_T5, random = ~1|year_T5/RING, data = age, na.action =
na.omit, method = "REML")
length_T5.model6f = lme(length_T5~sex+ WS_age + BS_age +
factor(year_T5) +weird_cat_T5, random = ~1|RING, data = age,
na.action = na.omit, method = "REML")
anova(length_T5.model6n, length_T5.model6r, length_T5.model6f)


# full model with gbar
length_T5.model1 = lme(length_T5~sex+ WS_age + BS_age +
factor(year_T5) +weird_cat_T5 + gbar, random = ~1|RING, data = age,
na.action = na.omit, method = "ML")
anova(length_T5.model6)


# full model - year
length_T5.model3 = update(length_T5.model1, .~.-factor(year_T5))
anova(length_T5.model1, length_T5.model3)

# full model - sex
```

```
length_T5.model4 = update(length_T5.model3, .~.-sex)
anova(length_T5.model3, length_T5.model4)

# full model - weird
length_T5.model5 = update(length_T5.model3, .~.-weird_cat_T5)
anova(length_T5.model3, length_T5.model5)

# full model - year
length_T5.model2 = update(length_T5.model3, .~.-gbar)
anova(length_T5.model1, length_T5.model2)


# full model where year_T5 considered as a variable
length_T5.model6 = lme(length_T5~sex+ WS_age + BS_age + year_T5
+weird_cat_T5 +gbar, random = ~1|RING, data = age, na.action =
na.omit, method = "ML")
anova(length_T5.model1, length_T5.model6)



anova(length_T5.model1, length_T5.model2, length_T5.model3,
length_T5.model4,length_T5.model5)


#best model with REML
best.length_T5 = lme(length_T5~sex+ WS_age + BS_age + weird_cat_T5 +
gbar, random = ~1|RING, data = age, na.action = na.omit, method =
"REML")
summary(best.length_T5)
anova(best.length_T5)

#best model with REML without gbar
besto.length_T5 = lme(length_T5~sex+ WS_age + BS_age +
factor(year_T5)+ weird_cat_T5, random = ~1|RING, data = age,
na.action = na.omit, method = "REML")
summary(besto.length_T5)
anova(besto.length_T5)

#------- mass_T5 general age effect
mass_T5.model_age = lme(mass_T5~age_ind_b, random = ~1|RING, data =
age, na.action = na.omit, method = "REML")
summary(mass_T5.model_age)
anova(mass_T5.model_age)

# full model random year_T5 REML
mass_T5.model11n = lme(mass_T5~sex+ WS_age + BS_age + weird_cat_T5,
random = ~1|RING, data = age, na.action = na.omit, method = "REML")
mass_T5.model11r = lme(mass_T5~sex+ WS_age + BS_age + weird_cat_T5,
random = ~1|year_T5/RING, data = age, na.action = na.omit, method =
"REML")
mass_T5.model11f = lme(mass_T5~sex+ WS_age + BS_age +
factor(year_T5)+weird_cat_T5, random = ~1|RING, data = age,
na.action = na.omit, method = "REML")
anova(mass_T5.model11n,mass_T5.model11r,mass_T5.model11f)
```

```
# full model with gbar
mass_T5.model6 = lme(mass_T5~sex+ WS_age + BS_age +
factor(year_T5)+weird_cat_T5 +gbar, random = ~1|RING, data = age,
na.action = na.omit, method = "ML")
anova(mass_T5.model6)

# full model - gbar
mass_T5.model7 = update(mass_T5.model6, .~.-gbar)
anova(mass_T5.model6, mass_T5.model7)

# full model - year
mass_T5.model8 = update(mass_T5.model6, .~.-factor(year_T5))
anova(mass_T5.model6, mass_T5.model8)

# full model - sex
mass_T5.model9 = update(mass_T5.model8, .~.-sex)
anova(mass_T5.model8, mass_T5.model9)

# - weird_cat_T5
mass_T5.model10 = update(mass_T5.model8, .~.-weird_cat_T5)
anova(mass_T5.model8, mass_T5.model10)

anova(mass_T5.model6, mass_T5.model7, mass_T5.model8,
mass_T5.model9, mass_T5.model10)

#best model with REML
best.mass_T5 = lme(mass_T5~sex+ WS_age + BS_age + weird_cat_T5 +
gbar, random = ~1|RING, data = age, na.action = na.omit, method =
"REML")
summary(best.mass_T5)
anova(best.mass_T5)


#------- width_T5 score general age effect
width_T5.modelage = lme(width_T5~age_ind_b, random = ~1|RING, data =
age, na.action = na.omit, method = "REML")
summary(width_T5.modelage)
anova(width_T5.modelage)

#full model random width_T5  REML
width_T5.model15n = lme(width_T5~sex+ WS_age + BS_age, random = ~1|
RING, data = age, na.action = na.omit, method = "REML")
width_T5.model15r = lme(width_T5~sex+ WS_age + BS_age, random = ~1|
year_T5/RING, data = age, na.action = na.omit, method = "REML")
width_T5.model15f = lme(width_T5~sex+ WS_age + BS_age +
factor(year_T5), random = ~1|RING, data = age, na.action = na.omit,
method = "REML")
anova(width_T5.model15n,width_T5.model15r,width_T5.model15f)


#full model for width_T5 with gbar
width_T5.model11 = lme(width_T5~sex+ WS_age + BS_age +
factor(year_T5) + gbar, random = ~1|RING, data = age, na.action =
```

```
na.omit, method = "ML")
summary(width_T5.model1)
anova(width_T5.model1)

width_T5.model12 = update(width_T5.model11, .~.-gbar)
anova(width_T5.model11, width_T5.model12)

width_T5.model13 = update(width_T5.model11, .~.-factor(year_T5))
anova(width_T5.model11, width_T5.model13)

width_T5.model14= update(width_T5.model13, .~. -sex )
anova(width_T5.model13, width_T5.model14)

width_T5.model15= update(width_T5.model14, .~. -gbar )
anova(width_T5.model14, width_T5.model15)

anova(width_T5.model11, width_T5.model12, width_T5.model13,
width_T5.model14, width_T5.model15)

#------- best model with REML
best.width_T5 = lme(width_T5~ WS_age + BS_age + gbar, random = ~1|
RING, data = age, na.action = na.omit, method = "REML")
summary(best.width_T5)
anova(best.width_T5)


#------------------------------------------------------------
#-------PCA1 score (vmx1) general age effect
vmx1.model_age = lme(vmx1~age_ind_b, random = ~1|RING, data = age,
na.action = na.omit, method = "REML")
summary(vmx1.model_age)
anova(vmx1.model_age)

# full model with gbar
vmx1.model4 = lme(vmx1~sex+ WS_age + BS_age + factor(year_T5) +
weird_cat_T5 + gbar, random = ~1|RING, data = age, na.action =
na.omit, method = "ML")
anova(vmx1.model4)

# full model - year
vmx1.model5 = update(vmx1.model4, .~.-factor(year_T5))
anova(vmx1.model4, vmx1.model5)

# full model - sex
vmx1.model6 = update(vmx1.model5, .~.-sex)
anova(vmx1.model5, vmx1.model6)

# full model - weird
vmx1.model7 = update(vmx1.model5, .~.-weird_cat_T5)
anova(vmx1.model5, vmx1.model7)

# full model - gbar
vmx1.model8 = update(vmx1.model5, .~.-gbar)
anova(vmx1.model5, vmx1.model8)
```

```
anova(vmx1.model4, vmx1.model5, vmx1.model6, vmx1.model7,
vmx1.model8)

#best model with REML
best.vmx1 = lme(vmx1~sex+ WS_age + BS_age + weird_cat_T5 + gbar,
random = ~1|RING, data = age, na.action = na.omit, method = "REML")
summary(best.vmx1)
anova(best.vmx1)

#------- PCA2 score (vmx2) general age effect
vmx2.modelage = lme(vmx2~age_ind_b, random = ~1|RING, data = age,
na.action = na.omit, method = "REML")
summary(vmx2.modelage)
anova(vmx2.modelage)

#full model for vmx2 with gbar
vmx2.model9 = lme(vmx2~sex+ WS_age + BS_age + factor(year_T5) +
gbar, random = ~1|RING, data = age, na.action = na.omit, method =
"ML")
summary(vmx2.model9)
anova(vmx2.model9)

vmx2.model10 = update(vmx2.model9, .~.-factor(year_T5))
anova(vmx2.model9, vmx2.model10)

vmx2.model11 = update(vmx2.model10, .~.-sex)
anova(vmx2.model10, vmx2.model11)

vmx2.model12 = update(vmx2.model11, .~.-gbar)
anova(vmx2.model11, vmx2.model12)

anova(vmx2.model9, vmx2.model10, vmx2.model11, vmx2.model12)

#------- for all data of model a
best.vmx2a = lme(vmx2~ WS_age + BS_age + gbar, random = ~1|RING,
data = age, na.action = na.omit, method = "REML")
summary(best.vmx2a)
anova(best.vmx2a)



#----BENDING
bending.modelage =  lme(bending_T5~age_ind_b, random = ~1|RING, data
= age, na.action = na.omit, method = "REML")
anova(bending.modelage)

# FULL model   with gbar
bending.model13 =  lme(bending_T5~vmx1 + vmx2 + sex + WS_age +
BS_age + factor(year_T5)+ gbar, random = ~1|RING, data = age,
na.action = na.omit, method = "ML")
summary(bending.model10)
anova(bending.model10)
```

```
bending.model14 = update(bending.model13, .~.-factor(year_T5))
anova(bending.model13, bending.model14)

bending.model15 = update(bending.model14, .~.-sex)
anova(bending.model14, bending.model15)

bending.model16 = update(bending.model15, .~.-gbar)
anova(bending.model15, bending.model16)

bending.model17 = update(bending.model16, .~.-vmx2)
anova(bending.model16, bending.model17)

bending.model18 = update(bending.model16, .~. -vmx1)
anova(bending.model16, bending.model18)

anova(bending.model13, bending.model14,bending.model15,
bending.model16, bending.model17, bending.model18)


# best REML    model 1
best.bending =  lme(bending_T5~vmx1 + vmx2 + WS_age + BS_age, random
= ~1|RING, data = age, na.action = na.omit, method = "REML")
summary(best.bending)
anova(best.bending)



# residuals bending REML
resid.bending =  lme(bending_T5~vmx1 + vmx2, random = ~1|RING, data
= age, na.action = na.omit, method = "REML")
resid2.bending =  lme(bending_T5~vmx1 + vmx2 + WS_age + BS_age,
random = ~1|RING, data = age, na.action = na.omit, method = "REML")
summary(resid.bending)
anova(resid.bending)

# FULL day extended model ML
day.model1 =  lme(day~ sex + WS_age + BS_age + factor(year_T5)+ vmx1
+ vmx2 + bending_T5 + gbar, random = ~1|RING, data = age, na.action
= na.omit, method = "ML")
summary(day.model1)
anova(day.model1)

day.model2 = update(day.model1, .~.-gbar)
anova(day.model1, day.model2)

day.model3 = update(day.model2, .~.-bending_T5)
anova(day.model2, day.model3)

day.model4 = update(day.model3, .~.-vmx2)
anova(day.model3, day.model4)

day.model5 = update(day.model4, .~.-vmx1)
anova(day.model4, day.model5)
```

```
day.model6 = update(day.model4, .~.-sex)
anova(day.model4, day.model6)

day.model7 = update(day.model6, .~.-factor(year_T5))
anova(day.model6, day.model7)

day.model8 = lme(day~ WS_age + BS_age + year_T5 + vmx1, random = ~1|
RING, data = age, na.action = na.omit, method = "ML")
anova(day.model6, day.model8)

anova(day.model1, day.model2, day.model3, day.model4, day.model5,
day.model6, day.model7, day.model8)

# best REML
best.day =  lme(day~ WS_age + BS_age + factor(year_T5) + vmx1,
random = ~1|RING, data = age, na.action = na.omit, method = "REML")
summary(best.day)
anova(best.day)



# ---- Outliers of growth bar
bpg = boxplot(bend$gbar)
bpg$out
# (<1.92, >3.02 outlier for growth bar

bend = bend[-which(bend$gbar > 2.98 | bend$gbar < 1.92),]

#-------gbar general age effect
gbar.modelage = lme(gbar~age_ind_b, random = ~1|RING, data = age,
na.action = na.omit, method = "REML")
summary(gbar.modelage)
anova(gbar.modelage)

# FULL model for all1b
gbar.model1 = lme(gbar~sex + WS_age + BS_age + factor(year_T5),
random = ~1|RING, data = age,  na.action = na.omit, method = "ML")
summary(gbar.model1)
anova(gbar.model1)

gbar.model2 = update(gbar.model1, .~.-factor(year_T5))
anova(gbar.model1, gbar.model2)

gbar.model3 = update(gbar.model2, .~.-sex)
anova(gbar.model2, gbar.model3)

anova(gbar.model1, gbar.model2, gbar.model3)

# best model REML
best.gbar = lme(gbar~ WS_age + BS_age , random = ~1|RING, data =
age,  na.action = na.omit, method = "REML")
summary(best.gbar)
anova(best.gbar)
```

```
#-------weirdness general age effect

# FULL GLM model for weirdness, binomial approach ????
ww.model16 = glm(weird_cat_T5~ sex + WS_age + BS_age + day + gbar +
factor(year_T5), data = age,  na.action = na.omit, family =
binomial)

summary(ww.model16)
anova(ww.model16)

ww.model17 = update(ww.model16, .~.-factor(year_T5))
anova(ww.model16, ww.model17)
summary(ww.model17)

ww.model18 = update(ww.model17, .~.-sex)
anova(ww.model17, ww.model18)
summary(ww.model18)

ww.model19 = update(ww.model18, .~.-gbar)
anova(ww.model18, ww.model19)
summary(ww.model19)

ww.model20 = update(ww.model19, .~.-day)
anova(ww.model19, ww.model20)
summary(ww.model20)

ww.model21 = update(ww.model18, .~. + bending_T5)
anova(ww.model19, ww.model21)
summary(ww.model21)


#----- Moultday
moultday.modelage = lme(moultday~age_ind_b, random = ~1|RING, data =
age, na.action = na.omit, method = "REML")
summary(moultday.modelage)
anova(moultday.modelage)

# FULL model
moultday.model21 = lme(moultday~sex + WS_age + BS_age +
factor(year_T5) +weird_cat_T5, random = ~1|RING, data = age,
na.action = na.omit, method = "ML")
summary(moultday.model21)
anova(moultday.model21)

moultday.model22 = update(moultday.model21, .~.- factor(year_T5))
anova(moultday.model21, moultday.model22)

moultday.model23 = update(moultday.model22, .~.- sex)
anova(moultday.model22, moultday.model23)


moultday.model24 = update(moultday.model23, .~.- weird_cat_T5)
anova(moultday.model23, moultday.model24)
```

```
anova(moultday.model21, moultday.model22, moultday.model23,
moultday.model24)


# best model for all data   model 1
best.moultday1 = lme(moultday~ sex + WS_age + BS_age + weird_cat_T5,
random = ~1|RING, data = age, na.action = na.omit, method = "REML")
summary(best.moultday1)
anova(best.moultday1)

# best model for all data   model 2
best.moultday2 = lme(moultday~ WS_age + BS_age + weird_cat_T5,
random = ~1|RING, data = age, na.action = na.omit, method = "REML")
summary(best.moultday2)
anova(best.moultday2)

#-------FIGURES --------  PLOTS
matched(length_T5~age+RING, data = age, label = F, xlab = "Age
(years)", ylab = "Length of feather (mm)")
matched(length_T5~year_T5+RING, data = age, label = F, xlab =
"Year", ylab = "Length of feather (mm)")

matched(mass_T5~age+RING, data = age, label = F, xlab = "Age
(years)", ylab = "Mass of feather (mg)")
matched(width_T5~age+RING, data = age, label = F, xlab = "Age
(years)", ylab = "Rachis diameter mm)")
matched(width_T5~year_T5+RING, data = age, label = F, xlab = "Age",
ylab = "Year ")

matched(vmx1~age+RING, data = age, label = F, ylab = "PC1 scores
(longitudinal size)", xlab = "Age (years)")
matched(vmx2~age+RING, data = age, label = F, ylab = "PC2 scores
(thickness)", xlab = "Age (years)")
matched(vmx2~year_T5+RING, data = age, label = F)

matched(gbar~age+RING, data = age, label = F, xlab = "Age (years)",
ylab = "Daily growth of feather (mm)")
matched(moultday~age+RING, data = age, label = F, ylab = "Duration
of moult (day)", xlab = "Age (year)")
matched(day~age+RING, data = age, label = F, xlab = "Age (years)",
ylab = "Day of capture (1= 1st of April)")
matched(day~year_T5+RING, data = age, label = F)

matched(bending_T5~age+RING, data = age, label = F, xlab = "Age
(years)", ylab = "Stiffness")

matched(residuals(resid.bending)~age+RING, data = age, label = F,
ylab = "Stiffness (residuals of model long. and vert. size of
feather)", xlab = "Age (year)")

matched(residuals(resid.length_T5)~year_T5+RING, data = age, label =
F, ylab = "Length(residuals of model length of feather)", xlab =
"Year")
matched(residuals(resid.length_T5)~popindexp+RING, data = age, label
```

```
= F, ylab = "Length(residuals of model length of feather)", xlab =
"Pop.index p")


matched(wing_length~age+RING, data = age, label = F)

boxplot(length_T5~age, data = age, ylab = "Length of feather (mm)",
xlab = "Age (years)")
boxplot(mass_T5~age, data = age, ylab = "Mass of feather (mg)", xlab
= "Age (years)")
boxplot(width_T5~age, data = age, ylab = "Rachis diameter (mm)",
xlab = "Age (years)")
boxplot(width_T5~year_T5, data = age, ylab = "Width T5 (mm)", xlab =
"Year")


boxplot(vmx1~age, data = age, ylab = "PC1 scores (longitudinal
size)", xlab = "Age (years)")
boxplot(vmx2~age, data = age, ylab = "PC2 scores (thickness)", xlab
= "Age (years)")
boxplot(vmx2~year_T5, data = age, ylab = "Width of the feather
(PCA2)", xlab = "Year")

boxplot(bending_T5~age, data = age, ylab = "Stiffness", xlab = "Age
(years)")

boxplot(gbar~age, data = age, ylab = "Daily growth of feather (mm)",
xlab = "Age (years)")
boxplot(moultday~age, data = age, ylab = "Duration of moult (day)",
xlab = "Age (years)")
boxplot(residuals(resid.bending)~age, data = age, ylab = "Stiffness
(residuals)", xlab = "Age (years)")
boxplot(residuals(resid2.bending)~year_T5, data = age, ylab =
"Stiffness (residuals of model age, size, width)", xlab = "Year")


boxplot(residuals(resid.length_T5)~year_T5, data = age, ylab =
"Length(residuals of model)", xlab = "Year")
boxplot(residuals(resid.length_T5)~popindexp, data = age, label= F,
ylab = "Length(residuals of model)", xlab = "Pop.index p")


boxplot(day~age, data = age, ylab = "Day of capture (1= 1st of
April)", xlab = "Age (years)")



plot(bending_T5~WS_age, data = age)
plot(bending_T5~BS_age, data = age)

par(mfrow = c(3,1), cex.lab = 1.4)
par(mai = c(0.4,0.6,0.1,0.2))
boxplot(vmx1~age, data = age, ylab = "PC1 scores", xaxt = "n")
par(mai = c(0.4,0.6,0.1,0.2))
```

```
boxplot(vmx2~age, data = age, ylab = "PC2 scores", xaxt = "n")
par(mai = c(0.6,0.6,0.1,0.2))
boxplot(bending_T5~age, data = age, ylab = "Stiffness", xlab =
"Age")


save.image("BendingResults29.RData")
```
