# Supplementary material for: Older birds have better feathers: A longitudinal study on the long-distance migratory Sand Martin, Riparia riparia
Source: PLoS One. 2019 Jan 4;14(1):e0209737. doi: 10.1371/journal.pone.0209737 (PMC6319700; doi:10.1371/journal.pone.0209737)
Supplement: S1 Dataset — (PDF) [file pone.0209737.s012.pdf]

RING	age_ind_b	age	sex	year_T5	bending_T5	width_T5	length_T5	mass_T5	weird_cat_T5	ALL1B	day	RINGER	wing_length	mass_g	tarsus	keel	tailm
L94241		3	M	1995	1.93718	0.77	58.7	6.58	1	2.4	95	0	106	13.6			53.5
L94241	4	4	M	1996	1.85007	0.75	58.4	6.80	0	2.3	89	K	106	13.0			
F39788		7	M	1995	2.04370	0.83	59.2	7.13	0	2.3	93	8	108	12.6			55.0
F80551		2	F	1995	2.00810	0.79	57.3	6.81	0	2.7	96	H	109	13.5			55.5
F80551	7	7	F	2000	1.86775	0.78	58.7	6.74	1	2.4	39	L	108	15.8		17.69	56.0
M10181		3	M	1996	2.01835	0.80	58.0	7.07	0	2.7	89	0	110	15.0			52.0
M10181	6	6	M	1999	2.35073	0.81	58.1	7.39	1	2.4	61	0	109	16.7		19.27	52.0
M31311		2	F	1997	1.80374	0.81	56.8	6.75	1	2.5	96	S	108	15.4			60.0
M31311	3	3	F	1998	1.88135	0.79	58.8	6.85	1	2.7	88	0	109	16.7		18.89	55.5
H74514		3	M	1997	1.98459	0.78	59.0	6.63	0	2.4	93	K	112	13.6			54.5
H74514	5	5	M	1999	2.22019	0.79	58.2	6.60	0	2.4	62	0	111	14.2		18.17	53.0
M57739		2	M	1997	2.27977	0.81	59.6	7.23	0	2.6	95	0	109	12.7		18.22	55.0
M57739	4	4	M	1999	2.20453	0.79	58.7	7.03	0	2.3	62	0	108	14.2		18.03	52.5
M57824		2	F	1997	2.68332	0.82	62.0	7.63	1	2.4	95	0	111	13.0		18.90	57.5
M57824	4	4	F	1999	2.96334	0.84	59.7	7.36	1	2.8	100	0	109	14.0		18.95	56.0
H73392		3	M	1997	2.36156	0.81	60.2	6.99	1	2.3	90	0	109	15.0		19.15	55.0
H73392	5	5	M	1999	2.59544	0.82	59.9	7.27	1	2.3	93	0	111	11.0		19.55	56.5
M33905		2	F	1998	2.40992	0.81	58.0	7.28	1	2.3	91	0	105	13.7		18.58	52.0
M33905	3	3	F	1999	2.23401	0.80	57.9	7.25	1	2.4	88	0	105	14.0		18.58	52.5
T12776		3	F	2000	2.43456	0.81	59.8	6.93	1	2.5	92	0	111	13.7	10.5	19.11	56.0
T12776	4	4	F	2001	2.60068	0.85	60.2	7.66	0	2.4	48	L	110	15.6		19.35	56.0
M33356		2	F	1998	2.20807	0.77	58.5	6.75	1	2.5	77	D	107	11.8			55.0
M33356	3	3	F	1999	2.28774	0.79	59.7	6.78	1	2.4	86	Z	107	12.0			55.5
L76608	6	6	F	1998	1.74063	0.81	57.3	6.77	0	2.4	88	K	107	14.1			55.0
T11607		2	M	1999	2.55266	0.88	59.2	7.16	0	2.7	86	0	110	12.4		18.78	53.0
T11607	5	5	M	2002	2.83205	0.86	59.3	7.35	0	2.0	63	L	112	14.3		18.78	55.0
L94183	7	7	F	1999	2.65062	0.83	59.6	7.80	1	2.4	93	P	109	13.1		19.00	56.0
T25334		2	M	1999	2.30682	0.83	57.7	6.88	0	2.1	93	0	108	13.5		18.56	53.0
T25334	3	3	M	2000	1.87537	0.82	56.5	6.50	0	2.3	96	L	109	13.2	10.1	18.51	56.0
H70297	5	5	M	1999	2.13912	0.79	60.6	7.45	0	2.4	88	Z	114	13.2			57.5
H71642	5	5	F	1999	2.29691	0.82	57.3	6.79	1	2.3	88	S	110	15.0			55.0
L95383	5	5	F	1997	2.13724	0.82	60.9	7.12	0	2.5	91	K	113	15.5			56.0
H71734		3	M	1997	1.87412	0.84	56.7	6.55	1	2.8	91	0	107	12.7		18.81	53.5
H71734	6	6	M	2000	1.57096	0.79	55.4	6.32	1	2.3	94	0	107	13.8	11.0	18.77	49.5
T11467		2	F	1999	2.12119	0.85	56.7	6.34	0	2.1	88	0	108	13.4		18.91	52.0
T11467	5	5	F	2002	1.73848	0.83	58.1	6.58	0	2.6	44	L	109	13.5		18.99	56.0
H70062	5	5	M	1999	2.79650	0.84	62.7	7.52	0	2.5	92	0	111	14.2		18.98	57.0
F81905	7	7	M	2000	1.98592	0.84	59.0	6.76	1	2.7	90	L	104	14.6	10.9	18.57	55.0
4E0475		1	F	2000	2.34931	0.84	58.9	6.94	1	2.2	89	L	107	13.8	10.0	18.64	55.5
4E0475	7	7	F	2006	2.77067	0.87	59.7	7.39	1	2.5	89	Z	110	14.4	10.0	18.52	57.0
T94690		1	M	2000	2.28850	0.81	60.8	6.86	0	2.3	89	L	110	13.2	10.5	18.74	58.0
T94690	2	2	M	2001	2.01526	0.81	59.9	6.46	1	2.5	95	0	111	14.0		18.70	55.0
T92584		2	M	2000	1.59571	0.80	53.4	5.67	1	2.7	73	L	107	13.6		18.43	55.0

T92584	4	4	M	2002	1.70692	0.80	54.8	6.03	1	2.5	54	L	109	13.1		18.51	50.0
T95541		1	M	2000	1.74325	0.80	55.3	6.43	1	2.6	74	L	106	12.8		18.75	50.0
T95541	3	3	M	2002	2.08109	0.79	57.1	6.39	1	2.4	52	L	110	12.3		19.07	53.0
H74875	6	6	M	2000	2.32986	0.86	56.7	6.70	1	2.1	90	L	105	14.1	9.8	18.39	52.0
4E1786	1	1	M	2001	2.01529	0.78	58.9	6.64	0	2.1	58	L	110	15.5		19.09	57.0
4E1786		2	M	2002	2.10130	0.76	59.0	6.78	0	2.2	44	L	113	13.4		19.21	55.0
T94961		1	F	2000	2.01668	0.85	56.9	6.42	1	2.2	73	L	107	15.5		18.07	54.5
T94961	2	2	F	2001	1.81067	0.84	58.7	6.86	0	2.5	60	L	107	14.9		17.92	54.5
M57542	5	5	M	2000	2.45548	0.80	59.9	7.65	1	2.5	67	L	112	15.0		19.62	56.0
T95827	1	1	F	2000	2.28099	0.83	53.9	6.41	1	2.3	91	0	106	12.7	11.0		49.5
T95827		2	F	2001	2.21924	0.84	54.9	6.33	1	2.1	101	L	106	14.3		18.62	53.5
4E1514		2	M	2002	1.78592	0.76	58.9	6.29	0	1.9	52	L	115	14.0		18.29	56.0
4E1514	3	3	M	2003	1.64666	0.75	56.6	6.17	0	2.3	108	0	112	14.3	11.2	18.50	55.5
T94579		1	F	2000	2.47930	0.84	61.7	7.35	0	2.5	88	L	112	13.1	10.4	18.77	60.0
T94579	3	3	F	2002	2.71301	0.85	64.2	7.57	1	2.7	67	L	115	14.8		18.81	60.0
H70832		6	F	2000	2.81288	0.86	61.8	7.75	0	2.7	53	L	110	15.5		18.74	57.0
H70832	7	7	F	2001	2.80359	0.84	59.2	7.19	1	2.4	97	0	109	14.6		18.23	54.0
T94630	1	1	M	2000	1.28705	0.76	53.0	5.31	1	2.5	98	Z	103	11.6	10.0	18.45	51.5
T94630		2	M	2001	1.35165	0.75	55.5	5.72	0	2.1	53	0	104	13.4		18.61	56.5
T65939		2	F	2000	2.57496	0.84	58.4	6.95	1	2.7	93	0	110	15.3	11.5	20.36	51.5
T65939	3	3	F	2001	2.44186	0.83	58.5	7.06	1	2.7	84	L	110	14.5		19.14	57.0
3E1829	1	1	F	2000	1.90110	0.83	56.9	6.30	0	2.2	94	0	108	14.0	10.8	20.73	52.0
3E1829		2	F	2001	2.22734	0.83	58.4	6.63	0	2.2	51	L	108	16.7		19.06	54.5
T142161	1	1	F	2001	1.63192	0.76	57.7	6.62	0	2.5	95	Z	107	13.1		18.73	
T142161		2	F	2002	1.52928	0.78	59.3	7.00	0	2.5	65	L	107	13.9		18.97	55.0
T144723		1	F	2001	2.21633	0.79	56.5	6.22	1	2.6	97	0	105	14.0		18.95	52.5
T144723	2	2	F	2002	2.16131	0.74	57.5	6.43	0	2.5	96	0	106	13.8		19.27	51.5
T92866		2	M	2001	1.84086	0.82	56.2	6.38	1	2.4	53	0	108	14.0		18.45	53.0
T92866	3	3	M	2002	1.86882	0.76	54.8	5.80	1	2.4	54	L	112	13.6		18.00	56.0
4E1086		2	M	2001	1.88829	0.79	55.5	5.89	1	2.6	55	L	105	14.2		17.68	52.0
4E1086	3	3	M	2002	1.42277	0.78	53.7	5.82	0	2.2	56	L	108	14.3		17.95	51.0
T144108		1	M	2001	1.66543	0.78	55.4	6.30	0	2.3	67	L	106	11.4		19.03	53.0
T144108	2	2	M	2002	1.94187	0.76	56.8	6.57	0	2.7	56	L	107	13.4		19.03	55.0
T95021		2	M	2001	2.10715	0.80	56.0	6.56	0	2.3	58	L	106	13.6		19.17	55.0
T95021	3	3	M	2002	1.53847	0.74	52.8	5.58	1	2.3	97	L	108	12.8		18.96	54.5
4E2869		2	M	2002	2.47313	0.83	58.2	6.83	0	2.7	47	L	111	13.9		18.57	57.0
4E2869	6	6	M	2006	2.69115	0.84	59.1	7.19	0	2.5	49	Z	112	13.7	10.7	18.07	58.0
T93928		2	F	2001	1.99503	0.79	55.6	6.51	1	2.3	94	0	103	15.4		19.41	51.0
T93928	3	3	F	2002	2.29320	0.81	57.8	7.13	0	2.6	48	L	107	16.0		19.74	55.0
T141183		1	M	2001	1.83691	0.79	57.3	6.25	0	2.4	90	Z	111	14.2		18.37	53.0
T141183	2	2	M	2002	2.30430	0.84	61.0	7.30	0	2.5	54	L	111	13.8		18.43	56.0
4E1757		1	M	2001	1.98950	0.80	58.8	6.70	1	2.1	76	L	111	13.6		19.45	56.0
4E1757	7	7	M	2007	2.38601	0.82	60.4	7.26	1	2.9	87	0	115	14.3	10.3	19.82	57.0
T140126	1	1	F	2001	2.17195	0.80	56.8	6.36	1	2.6	91	L	109	15.4		18.90	

T140126		2	F	2002	2.07212	0.82	57.8	6.66	1	2.6	52	L	109	15.1		18.79	58.0
H74510	7	7	M	2001	2.49224	0.84	61.0	7.00	1	2.6	95	L	116	12.8		17.91	58.0
T217941		2	F	2003	2.33612	0.80	60.0	7.14	0	2.5	64	L	108	13.5		18.37	57.0
T217941	3	3	F	2004	2.42146	0.79	61.1	7.44	0	2.6	53	0	107	13.5	10.3	18.81	56.0
4E2587		1	M	2001	1.42300	0.79	55.9	5.63	1	2.2	50	L	105	13.1		17.86	52.0
4E2587	2	2	M	2002	2.01723	0.82	57.8	6.09	0	2.4	48	L	107	15.3		18.48	54.0
T146088	1	1	F	2001	2.01100	0.79	56.5	6.53	0	2.3	50	L	106	13.4		18.56	55.5
T146088		2	F	2002	2.10854	0.81	57.7	6.59	0	2.3	52	L	107	13.6		18.57	55.0
T142335		1	F	2001	1.80896	0.78	57.4	6.56	0	2.4	85	L	106	13.5		18.75	54.5
T142335	2	2	F	2002	1.82199	0.80	57.9	6.90	0	2.5	73	L	108	12.5		18.72	55.0
4E1764		1	M	2001	2.04995	0.77	57.0	6.24	0	2.3	69	L	108	13.6		19.12	56.0
4E1764	2	2	M	2002	2.17242	0.77	58.9	6.48	1	2.4	92	L	111	13.3		19.15	56.0
T144283		1	F	2001	1.93249	0.79	59.6	6.76	0	2.4	92	L	111	13.1		18.03	
T144283	2	2	F	2002	2.15913	0.79	59.6	6.96	0	2.5	50	L	110	13.7		17.97	53.0
4E2573		1	M	2001	1.82375	0.84	56.9	6.08	1	2.6	92	L	110	13.1		19.08	56.0
4E2573	2	2	M	2002	2.47531	0.84	60.4	7.19	0	2.7	58	L	112	14.1		19.23	56.5
T147020		1	F	2001	2.16178	0.78	61.1	6.79	0	2.4	90	0	110	14.0		18.45	58.0
T147020	2	2	F	2002	2.40370	0.78	62.6	7.28	0	2.4	88	L	115	12.6		18.25	60.0
4E1051		2	M	2001	2.77953	0.88	61.2	7.68	0	2.5	55	Z	113	15.3		19.68	56.0
4E1051	3	3	M	2002	1.88050	0.87	60.1	7.47	1	2.6	59	L	113	15.0		18.45	56.5
T144156	1	1	F	2001	2.68951	0.79	56.3	6.36	0	2.1	98	L	108	13.2		18.41	55.5
T144156		2	F	2002	1.82669	0.79	58.5	6.37	0	2.3	56	L	109	14.7		18.54	55.0
T142068		1	M	2001	1.60883	0.78	56.0	6.39	1	2.3	92	L	107	13.0		17.67	53.5
T142068	2	2	M	2002	2.17833	0.79	59.0	7.09	0	2.6	46	L	110	13.4		17.93	57.0
H73504	7	7	M	2001	2.30624	0.83	58.6	6.95	1	2.9	85	L	109	13.2		18.99	56.0
T143080		2	M	2002	2.37497	0.82	59.3	7.11	0	2.6	94	0	109	13.5		19.59	54.5
T143080	3	3	M	2003	1.87757	0.76	57.7	6.31	0	2.1	64	L	114	12.8		17.67	55.0
T140057		2	F	2002	2.10499	0.82	59.7	7.34	1	2.7	91	L	110	13.2		18.36	58.0
T140057	3	3	F	2003	2.36899	0.79	59.3	7.51	0	2.7	82	0	107	13.4	10.2	18.89	56.0
T220678		1	M	2002	1.94300	0.79	60.6	6.29	1	2.5	97	0	111	13.1		19.32	54.5
T220678	2	2	M	2003	2.06821	0.81	61.6	6.59	1	2.1	87	0	112	12.3	10.1	19.77	58.0
T25364	5	5	M	2002	2.25453	0.86	60.5	7.17	0	2.6	96	0	107	13.8		19.35	57.0
T147450		1	M	2002	1.74061	0.76	54.3	5.62	1	2.4	88	L	107	12.1		18.41	53.0
T147450	3	3	M	2004	1.70306	0.76	55.3	5.71	0	2.1	59	0	106	12.6	10.4	19.17	51.5
T216087	1	1	M	2002	1.44792	0.78	59.6	6.37	0	2.8	74	L	112	14.7		18.58	57.5
T216087		2	M	2003	1.62093	0.71	57.7	6.12	1	2.2	56	L	113	15.3		18.58	59.0
T216864		1	F	2002	1.81242	0.78	56.5	5.95	1	2.8	89	L	110	12.6		18.84	51.0
T216864	2	2	F	2003	1.83986	0.79	58.2	6.23	0	2.2	56	Z	109	15.0		19.18	53.0
T217995	1	1	M	2002	1.56684	0.81	57.2	6.17	0	2.3	73	L	109	15.5		18.80	55.0
T217995		2	M	2003	1.93731	0.83	59.9	7.03	0	2.7	39	Z	111	15.1	10.8	18.67	57.5
T13000		5	F	2002	2.05461	0.86	59.5	6.83	1	2.5	73	L	112	12.8		18.40	57.0
T13000	6	6	F	2003	2.28020	0.82	58.5	6.75	1	2.5	87	Z	111	13.7	10.8	18.84	54.0
T142487		2	M	2002	1.67697	0.86	57.3	6.70	1	2.6	90	0	107	13.8		19.31	51.5
T142487	3	3	M	2003	2.00891	0.85	57.9	7.07	0	2.3	85	0	108	14.0	10.5	19.32	53.5

T215884		1	M	2002	1.96714	0.82	56.6	6.51	0	3.0	93	L	104	12.7		18.16	54.0
T215884	4	4	M	2005	1.94792	0.79	54.3	6.11	1	2.2	96	0	107	13.2	9.6	18.85	52.0
T216142		1	M	2002	1.55888	0.77	58.0	6.06	0	2.3	93	L	109	12.5		18.42	53.0
T216142	2	2	M	2003	1.62958	0.76	59.2	6.65	0	2.5	84	0	109	12.1	10.5	18.73	54.0
T216073		1	M	2002	1.67420	0.77	57.6	6.22	0	2.5	67	L	110	14.5		18.78	54.0
T216073	2	2	M	2003	1.66010	0.77	59.2	6.25	1	2.5	50	L	111	13.8	10.3	18.89	55.0
T93778		3	M	2002	2.56402	0.79	62.9	7.90	1	2.7	54	L	115	15.6		19.60	58.0
T93778	5	5	M	2004	2.45859	0.80	62.0	7.94	1	2.3	84	0	110	15.1	10.7	20.42	55.0
T297320		1	F	2003	1.73121	0.80	57.5	6.41	0	2.3	64	L	109	14.3		18.44	55.0
T297320	2	2	F	2004	2.24006	0.81	59.4	6.54	0	2.2	55	0	109	15.4	10.2	18.84	56.0
T297071	1	1	M	2003	1.53103	0.79	57.3	6.42	0	2.4	64	L	109	14.7		18.73	54.0
T297071		2	M	2004	1.98327	0.80	58.9	6.60	1	2.5	83	0	108	13.6	10.8	19.83	55.0
T296704		1	M	2003	2.20730	0.83	57.9	6.57	0	2.5	58	L	110	13.4		17.07	54.5
T296704	2	2	M	2004	2.10794	0.82	57.2	6.55	0	2.0	61	Z	109	13.0	9.9	16.76	54.0
T218908	1	1	M	2003	1.50659	0.78	53.4	5.41	1	2.6	89	0	104	12.0	10.2	19.08	49.0
T218908		2	M	2004	1.11248	0.75	53.8	5.76	1	2.3	55	0	105	12.7	10.2	19.37	49.0
T218800		1	F	2003	2.25879	0.86	57.3	6.87	1	2.6	88	Z	105	13.5	10.2	18.74	53.0
T218800	2	2	F	2004	2.03209	0.84	58.2	7.40	0	2.5	90	Z	108	12.7	10.1	18.72	55.0
T296002		1	F	2003	2.26457	0.84	55.3	6.48	1	2.6	84	Z	103	14.6	10.1	17.86	51.0
T296002	3	3	F	2005	1.94130	0.82	55.8	6.32	1	2.5	84	0	103	14.5	10.1	18.42	51.0
T297557	1	1	F	2003	1.72766	0.80	56.2	5.76	1	2.5	85	Z	106	13.9	11.2	18.57	53.0
T297557		2	F	2004	1.97162	0.78	59.1	6.22	1	2.4	85	0	107	13.5	11.4	18.98	53.5
T141857		3	M	2003	2.64601	0.82	61.2	7.67	0	2.6	87	Z	110	14.8	10.7	17.92	58.0
T141857	4	4	M	2004	2.66997	0.83	61.6	7.50	0	2.5	85	0	110	14.6	10.7	18.57	55.5
T297633		1	M	2003	2.10139	0.80	57.6	6.83	0	2.2	81	0	106	13.8	10.5	19.50	53.0
T297633	4	4	M	2006	2.07591	0.80	57.2	7.03	1	2.2	111	0	105	13.8	10.0	19.48	52.0
T296437		1	F	2003	2.31496	0.85	59.1	6.58	0	2.7	85	0	106	12.5	11.0	18.20	54.5
T296437	6	6	F	2008	2.57709	0.85	61.7	7.05	0	2.8	24	0	108	14.1	10.8	18.35	56.5
T296280		1	M	2003	2.39755	0.80	59.5	6.75	0	2.4	83	0	106	13.2	10.3	18.46	55.0
T296280	2	2	M	2004	2.26843	0.84	60.1	6.88	0	2.2	56	0	107	14.0	10.4	18.41	55.0
T296998		1	F	2003	2.30028	0.82	60.3	7.33	1	2.7	83	0	109	13.2	10.1	18.26	55.0
T296998	2	2	F	2004	2.36884	0.82	61.8	7.01	0	2.6	56	0	109	13.8	10.1	18.75	57.0
T147432		2	M	2003	1.52960	0.71	53.7	5.74	1	2.4	83	0	106	12.7	10.7	19.32	49.5
T147432	4	4	M	2005	1.29689	0.72	55.9	5.92	1	2.3	94	0	105	12.9	10.6	19.39	53.0
T297084		1	F	2003	1.46023	0.78	56.2	6.24	1	2.3	86	0	103	15.2	11.3	19.25	50.0
T297084	2	2	F	2004	1.61072	0.79	57.7	6.68	0	2.4	84	Z	106	15.2	10.6	18.72	53.0
T220086		2	F	2003	1.96162	0.80	61.5	7.35	0	2.4	81	0	108	14.0	10.6	18.43	57.0
T220086	3	3	F	2004	2.24994	0.79	62.3	7.61	1	2.2	99	Z	112	14.5		18.02	59.0
T296357	1	1	M	2003	1.67886	0.79	60.4	6.51	0	2.4	64	0	109	12.9	10.4	18.87	56.5
T296357		2	M	2004	1.86342	0.78	62.4	6.83	0	2.5	56	0	111	14.0	10.4	19.67	60.0
T218374		1	M	2003	1.90640	0.81	56.9	6.43	1	2.5	85	0	110	13.3	10.3	19.76	52.5
T218374	2	2	M	2004	2.12711	0.81	58.8	6.96	0	2.6	63	Z	112	14.5	9.9	18.35	55.5
T220056		2	M	2003	1.72421	0.80	58.1	6.21	1	2.8	84	0	106	11.7	10.3	17.88	52.0
T220056	3	3	M	2004	1.82111	0.80	59.6	6.52	1	2.8	100	0	105	12.7	10.2	17.84	52.0

T296722		2	M	2004	2.64372	0.83	58.7	7.01	1	2.9	80	Z	111	14.2	10.5	19.01	53.5
T296722	3	3	M	2005	2.96157	0.83	59.7	7.48	0	2.3	33	0	110	14.7	10.7	19.67	54.0
T297362		2	M	2004	2.25029	0.82	58.9	6.44	1	2.4	59	0	109	12.7	10.1	18.70	54.0
T297362	4	4	M	2006	1.85698	0.84	58.0	6.62	1	2.5	111	0	108	12.3	10.3	18.71	53.0
T375805		1	F	2004	2.31602	0.84	58.6	7.08	0	2.8	59	0	109	13.9	11.1	18.60	54.5
T375805	4	4	F	2007	2.42240	0.84	59.4	7.24	0	2.4	65	Z	110	15.0	10.7	17.86	58.0
T302865		1	M	2004	2.20465	0.86	60.6	7.34	0	2.9	56	0	113	13.4	11.0	19.14	56.0
T302865	2	2	M	2005	2.25552	0.82	59.6	7.28	0	2.4	67	Z	116	14.2	10.9	19.11	59.0
T304069		1	F	2004	1.85548	0.78	57.7	6.69	0	2.5	84	0	109	14.6	10.8	17.49	56.5
T304069	5	5	F	2008	2.02476	0.80	59.8	7.37	1	2.3	86	Z	110	14.4	10.6	17.79	58.5
3E1652	5	5	M	2004	2.64742	0.80	60.7	7.15	1	2.6	84	Z	110	13.1	10.6	18.13	57.0
4E2648		4	M	2004	2.40533	0.82	60.3	7.19	0	2.7	86	Z	109	13.4	10.4	18.73	59.0
4E2648	5	5	M	2005	2.28532	0.81	60.2	7.37	0	2.4	93	0	108	14.2	10.5	19.26	56.0
T218606		3	M	2004	2.82042	0.89	61.5	7.56	1	2.8	61	Z	115	14.9	10.7	20.27	58.0
T218606	5	5	M	2006	2.98478	0.86	62.0	7.88	1	2.4	55	Z	116	14.6	10.9	20.39	60.5
T303384		1	M	2004	1.86629	0.77	56.3	6.14	1	2.6	63	Z	109	13.2	10.4	19.00	51.5
T303384	3	3	M	2006	1.92497	0.77	55.8	6.20	1	2.4	110	Z	110	13.0	10.5	18.11	53.0
T299839		2	F	2004	1.98261	0.81	58.7	6.88	1	2.7	84	Z	111		10.2	18.88	56.0
T299839	4	4	F	2006	2.02426	0.81	59.4	6.58	1	2.7	76	Z	110	15.8	10.6	18.91	58.0
T296772		2	M	2004	2.22684	0.82	58.0	6.61	1	2.4	79	Z	106	13.1	10.2	19.95	53.5
T296772	4	4	M	2006	2.16682	0.84	58.2	6.94	0	2.6	77	Z	108	12.9	10.5	18.88	56.0
T304114		1	F	2004	2.37599	0.80	59.0	6.91	1	2.6	70	0	108	14.3	10.9	19.01	54.5
T304114	3	3	F	2006	2.21071	0.82	59.7	7.11	1	2.6	89	Z	110	15.2	10.5	18.45	59.5
T303955		1	M	2004	1.71634	0.77	58.4	6.03	1	2.6	79	Z	111	14.2	10.6	19.51	53.5
T303955	4	4	M	2007	1.80709	0.77	60.1	6.30	1	2.6	73	Z	114	13.5	10.5	18.97	57.0
T302988		1	M	2004	1.54783	0.78	56.7	5.88	0	2.3	56	0	108	11.9	10.5	18.89	52.0
T302988	3	3	M	2006	1.63581	0.79	57.7	6.01	1	2.6	90	0	109	12.7	10.2	18.28	54.0
T140385	5	5	M	2005	2.10567	0.86	60.2	7.29	1	2.5	93	0	110	13.2	10.9	19.48	54.0
T95646	6	6	F	2005	1.94386	0.78	58.9	6.74	1	2.3	96	0	112	14.6	10.8	19.23	52.5
T302512		2	M	2005	2.17931	0.83	55.8	6.31	0	2.4	98	0	106	12.5	10.5	19.39	53.0
T302512	3	3	M	2006	2.21642	0.83	56.8	6.55	0	2.4	109	Z	107	12.3	10.4	18.39	53.5
T304825	3	3	M	2006	2.17964	0.79	60.3	6.84	1	2.3	67	Z	110	14.3	10.8	19.52	58.0
T304276		2	M	2005	2.28104	0.86	58.2	6.99	0	2.3	100	0	109	13.6	10.4	20.58	52.0
T304276	5	5	M	2008	2.73579	0.87	60.0	7.13	1	2.5	81	0	110	13.7	10.8	19.56	55.0
T148177	5	5	F	2005	2.00981	0.83	57.7	6.63	1	2.2	99	Z	107	12.2	10.5	18.46	57.0
T304826		2	M	2005	2.18200	0.81	54.0	6.43	1	2.2	99	Z	105	12.2	10.5	18.20	56.0
T304826	4	4	M	2007	2.25245	0.85	58.2	7.01	1	2.2	91	0	105	12.5	10.3	18.34	52.0
T303216		2	F	2005	1.95236	0.81	57.3	6.11	1	2.0	100	Z	108	12.8	9.8	19.23	55.0
T303216	4	4	F	2007	1.97399	0.81	58.6	6.58	1	2.1	96	Z	111	11.6	9.8	18.21	57.5
T379925		1	M	2005	2.16738	0.82	54.9	6.29	1	2.1	93	0	107	13.5	10.1	19.08	52.5
T379925	5	5	M	2009	2.36487	0.82	59.1	7.35	1	2.2	65	0	109	13.7	10.5	18.89	55.0
T303676		3	F	2006	1.93933	0.79	59.9	6.67	1	2.3	48	Z	114	15.7	10.5	18.58	58.5
T303676	5	5	F	2008	2.16168	0.80	61.3	7.32	0	2.4	54	0	113	14.5	10.8	19.39	56.0
T303710		3	F	2006	2.41772	0.83	59.5	7.32	1	2.5	57	Z	112	15.1	10.3	19.34	57.0

T303710	5	5	F	2008	2.63056	0.84	59.2	7.20	0	1.9	24	0	110	14.1	10.6	19.65	54.5
T435050	1	1	F	2006	2.46348	0.85	62.7	7.66	0	2.6	67	Z	115	13.6	11.0	18.93	60.0
T435050		2	F	2007	2.45790	0.85	63.8	7.57	0	2.5	86	0	112	15.0	11.1	19.42	59.0
T431993		1	M	2006	2.16273	0.80	57.1	6.50	1	2.5	68	Z	108	13.7	10.9	20.03	56.0
T431993	3	3	M	2008	2.50442	0.84	58.7	6.73	1	2.6	62	0	107	13.4	10.5	19.00	56.0
T433650		1	M	2006	1.67925	0.74	56.5	5.66	1	2.4	88	0	106	13.5	10.4	19.28	52.0
T433650	3	3	M	2008	1.86316	0.78	58.7	6.10	0	2.5	62	0	108	13.1	9.9	19.55	54.0
T435268		1	M	2006	1.75524	0.78	52.7	5.48	1	2.2	89	0	103	13.2	10.5	20.04	50.5
T435268	2	2	M	2007	1.98914	0.79	58.2	6.75	1	2.6	89	0	106	11.7	10.5	20.07	53.0
T215941	5	5	M	2006	2.69060	0.79	60.2	6.76	1	2.7	93	0	113	14.7	10.6	19.11	56.0
T431968		1	M	2006	1.61111	0.82	55.3	6.32	1	2.5	74	Z	107	12.9	10.9	18.99	53.0
T431968	4	4	M	2009	2.11516	0.81	56.3	6.50	1	2.3	90	0	106	13.0	11.5	19.17	51.0
T375816		3	F	2006	2.30866	0.84	57.7	6.67	0	2.4	75	Z	112	14.6	10.5	19.52	56.5
T375816	5	5	F	2008	2.33738	0.83	57.4	6.66	1	2.3	87	0	111	15.4	10.9	20.11	53.0
T431881		1	F	2006	1.77227	0.77	56.3	6.37	1	2.0	76	Z	106	15.6	9.7	17.59	56.0
T431881	2	2	F	2007	1.94217	0.75	56.7	6.52	0	2.3	85	Z	107	13.4	10.1	16.94	56.5
T433217		1	F	2006	1.79296	0.78	56.8	6.27	1	2.5	82	Z	109	14.6	10.4	18.83	53.0
T433217	3	3	F	2008	2.00545	0.82	58.2	6.70	0	2.5	25	0	109	15.0	10.6	18.82	54.0
T435161		1	M	2006	1.63946	0.78	57.3	5.78	1	2.7	89	Z	109	15.1	10.2	18.71	54.5
T435161	2	2	M	2007	1.85154	0.80	58.5	6.24	0	2.3	104	Z	111	12.8	10.1	18.73	58.5
T432503	1	1	M	2006	1.86063	0.80	55.4	6.03	0	2.4	91	Z	110	13.2	11.0	18.86	54.5
T432503		2	M	2007	1.86433	0.81	56.9	6.30	0	2.4	89	Z	112	12.6	11.0	18.60	56.0
T433204		1	F	2006	1.62118	0.74	55.6	5.94	1	2.2	93	Z	110	12.6	10.5	18.37	54.0
T433204	2	2	F	2007	1.81421	0.72	56.7	6.40	0	2.8	88	Z	112	14.3	10.2	18.22	56.5
T431906		1	M	2006	1.36175	0.78	54.6	6.05	1	2.4	109	Z	108	12.9	9.8	19.01	52.5
T431906	3	3	M	2008	1.68822	0.79	56.0	6.51	1	2.0	84	Z	108	12.8	9.7	18.73	54.5
T432718		1	F	2006	2.47243	0.88	58.5	8.23	1	2.4	109	Z	112	13.1	11.3	19.03	55.5
T432718	2	2	F	2007	2.49316	0.82	59.8	7.47	1	2.7	86	0	112	13.8	11.4	19.62	54.5
T150537	5	5	M	2006	1.46744	0.71	56.3	5.49	1	2.2	108	0	108	11.6	10.6	18.33	53.5
T431545		2	M	2006	1.82556	0.80	56.2	6.17	1	2.4	108	0	104	11.4	10.1	19.13	52.0
T431545	5	5	M	2009	2.03700	0.80	56.3	6.29	1	2.1	65	0	105	12.7	10.7	19.93	51.0
T375367		3	M	2006	2.13065	0.85	59.5	6.90	1	2.6	109	0	110	11.3	10.8	18.41	54.0
T375367	5	5	M	2008	2.49435	0.84	59.8	6.84	1	2.4	62	0	109	12.8	10.5	19.23	54.5
T431818		2	F	2007	1.73499	0.78	58.2	6.16	0	2.4	65	Z	113	16.4	10.8	18.42	58.0
T431818	3	3	F	2008	1.73117	0.77	58.6	6.45	0	2.3	61	Z	111	17.2	10.8	18.45	55.5
W05876		1	M	2007	1.77046	0.79	57.0	6.10	0	2.5	65	Z	108	13.1	10.2	17.34	54.0
W05876	2	2	M	2008	1.80457	0.76	58.0	6.13	0	2.4	88	Z	108	12.0	10.3	17.59	57.0
T150594	6	6	M	2007	2.37530	0.85	55.9	7.03	0	2.5	66	Z	111	15.1	11.2	19.04	57.0
T297207	5	5	F	2007	2.29380	0.81	59.7	7.00	1	2.7	87	Z	111	12.9	11.0	17.67	60.0
T432688		2	M	2007	1.80417	0.76	58.1	6.29	0	2.5	88	Z	112	11.1	9.8	17.71	57.0
T432688	4	4	M	2009	1.82251	0.77	58.6	6.61	0	2.4	20	0	108	12.3	9.8	18.45	59.0
W05847		1	M	2007	2.43279	0.93	56.2	6.89	1	2.3	88	Z	106	12.6	10.4	17.15	53.5
W05847	2	2	M	2008	2.25223	0.92	57.7	7.18	1	2.4	84	Z	105	14.3	10.3	17.42	53.0
T431598		3	M	2007	3.03263	0.85	58.9	7.27	1	2.6	85	0	112	12.8	10.5	18.21	53.0

T431598	4	4	M	2008	2.87364	0.87	59.0	6.98	1	2.6	83	Z	113	12.5	10.8	18.46	56.0
T433058		2	M	2007	2.34665	0.83	59.9	7.07	1	2.6	88	0	112	13.2	10.8	18.94	55.0
T433058	4	4	M	2009	2.33303	0.84	57.5	6.61	1	2.4	69	Z	113	12.7	10.8	18.92	56.5
T433900		2	F	2007	2.22987	0.81	60.4	6.83	1	2.4	90	0	111	13.7	10.7	19.33	54.0
T433900	3	3	F	2008	1.92553	0.80	60.7	6.98	0	2.1	46	0	109	15.4	10.5	19.58	55.0
T432687		2	F	2007	2.43200	0.80	58.8	6.80	1	2.5	97	Z	112	14.6	10.9	18.94	58.5
T432687	3	3	F	2008	1.85713	0.78	57.8	7.16	0	2.4	51	0	110	14.3	11.2	19.55	52.0
T302335		5	F	2008	2.94453	0.82	60.2	7.86	0	2.5	21	0	108	14.0	10.5	19.00	56.5
T302335	7	7	F	2010	3.11563	0.84	61.4	8.09	1	2.4	94	0	107	14.0	10.3	19.48	55.0
W07863		1	M	2008	1.86935	0.79	57.3	6.65	0	2.5	25	0	110	14.7	10.6	18.44	51.5
W07863	2	2	M	2009	2.09665	0.80	59.0	6.84	1	2.6	49	0	111	14.0	10.6	18.68	54.0
T303847	5	5	F	2008	2.93441	0.86	62.1	7.55	1	2.7	26	0	106	15.2	10.8	18.56	57.0
T218891	6	6	M	2008	2.62915	0.85	58.8	7.03	0	2.7	44	0	106	14.3	10.5	19.90	54.0
W06397		2	M	2008	2.32069	0.78	57.9	6.51	0	2.5	44	0	112		11.3	17.61	56.0
W06397	3	3	M	2009	1.90324	0.78	59.0	6.92	0	2.3	94	0	111	12.6	11.0	18.27	55.0
W07448	1	1	M	2008	2.75310	0.83	60.3	7.28	0	2.7	44	0	109	14.2	10.9	19.98	55.0
W07448		2	M	2009	2.69580	0.82	60.8	7.67	1	2.7	72	Z	112	14.2	10.6	19.64	58.0
T433273		3	M	2008	2.51564	0.84	60.2	7.40	0	2.6	44	0	109	15.3	10.5	20.02	55.5
T433273	4	4	M	2009	2.29404	0.82	59.2	6.86	1	2.7	92	Z	109	13.7	10.5	19.52	57.5
T301987	5	5	F	2008	2.45078	0.85	60.8	6.84	0	2.3	61	0	114	15.1	10.6	19.55	54.5
W08440		1	M	2008	1.89455	0.83	57.4	6.70	0	2.1	84	0	112	13.4	10.3	17.99	56.0
W08440	3	3	M	2010	2.69959	0.87	61.4	7.72	1	2.5	94	0	111	13.8	10.6	18.04	58.0
W09656		1	M	2008	2.02047	0.81	57.8	6.62	0	2.3	86	0	108	13.2	11.0	18.82	53.0
W09656	2	2	M	2009	1.95364	0.81	58.0	7.04	0	2.1	69	Z	108	13.6	10.6	18.38	57.5
W07942		1	F	2008	1.88620	0.81	60.6	6.74	1	2.6	86	0	112	13.6	10.2	18.66	56.0
W07942	3	3	F	2010	1.87803	0.82	59.3	6.47	1	2.4	83	Z	109	13.8	10.2	18.92	55.0
W08026		1	F	2008	2.21421	0.84	60.2	7.02	1	2.5	87	0	109	12.9	10.5	18.19	55.0
W08026	3	3	F	2010	2.46400	0.84	60.4	7.47	1	2.4	70	Z	106	16.0	10.6	17.96	57.0
W09019		1	M	2008	1.53097	0.76	58.4	5.85	1	2.2	88	0	109	14.6	10.7	19.64	53.0
W09019	3	3	M	2010	1.96779	0.81	59.8	6.56	1	2.3	95	Z	110	14.6	11.0	19.52	59.0
T446378		2	M	2008	1.70073	0.82	55.3	6.50	1	2.6	83	Z	104	12.8	10.8	17.79	57.0
T446378	3	3	M	2009	1.86556	0.81	57.2	6.79	1	2.3	90	0	104	13.0	10.8	18.20	52.0
W09497		1	M	2008	2.23177	0.83	59.5	7.30	0	2.7	83	Z	113	12.6	10.6	19.03	59.0
W09497	3	3	M	2010	2.20236	0.79	56.4	6.83	1	2.5	100	Z	111	12.2	10.7	18.74	59.0
T304981	5	5	M	2008	1.73701	0.84	55.0	6.38	1	2.3	84	Z	105	12.6	10.3	18.44	51.0
W09395	1	1	F	2008	1.56556	0.78	56.6	5.70	0	2.7	84	Z	108	12.9	10.5	18.22	56.0
W09395		2	F	2009	1.71714	0.73	58.2	5.91	0	2.8	88	0	107	12.3	10.9	19.11	55.0
W45559		1	M	2008	1.50719	0.78	54.6	5.94	0	2.4	85	Z	107	0.0	9.8	18.60	52.5
W45559	2	2	M	2009	1.85976	0.79	55.7	6.21	1	2.5	72	Z	105	13.4	9.7	18.36	53.5
T302994	5	5	M	2008	2.38053	0.82	57.9	6.71	1	2.6	87	Z	109	12.9	10.3	17.18	57.5
T218899	6	6	F	2008	2.21121	0.81	59.5	6.88	1	2.1	87	Z	107	13.3	10.7	18.85	58.5
T434471		3	M	2008	1.70763	0.81	57.6	6.85	1	2.5	87	Z	108	14.0	10.3	18.70	56.0
T434471	4	4	M	2009	1.89630	0.79	56.1	6.77	0	2.1	94	0	106	13.4	10.4	18.92	52.0
W09392		1	M	2008	2.20208	0.84	54.9	6.20	0	2.3	87	Z	107	13.8	10.6	18.33	55.0

W09392	2	2	M	2009	2.05322	0.85	55.3	6.43	1	2.4	94	Z	106	13.6	10.4	18.42	54.0
T378111	5	5	M	2009	2.32563	0.80	59.7	7.15	0	2.2	46	0	109	15.3	9.7	19.48	55.0
W47335	1	1	F	2009	1.80380	0.78	58.9	6.36	1	2.4	70	0	107	14.8	10.9	18.56	55.0
W47335		2	F	2010	1.77495	0.77	60.1	6.65	0	2.4	86	Z	108	14.6	10.9	17.89	59.0
W47512		1	M	2009	1.95621	0.79	56.9	6.29	0	2.3	70	0	115	13.7	10.6	20.42	53.0
W47512	2	2	M	2010	2.26669	0.79	57.6	6.43	0	2.4	70	Z	106	13.1	10.3	17.98	55.0
W47540		1	M	2009	2.12163	0.79	59.4	7.14	1	2.5	88	0	110	13.0	10.4	19.29	54.0
W47540	2	2	M	2010	2.34425	0.79	59.8	7.06	1	2.4	99	Z	111	13.0	10.5	18.11	59.0
W48729	1	1	F	2009	1.97508	0.78	57.6	6.64	1	2.5	91	0	109	14.0	10.5	19.02	53.0
W48729		2	F	2010	2.51102	0.80	59.2	7.20	1	2.6	101	Z	109	13.6	10.8	18.25	56.5
W49900		1	F	2009	1.46010	0.80	54.5	6.25	1	2.3	87	Z	107	14.2	10.9	18.49	53.5
W49900	2	2	F	2010	1.45924	0.79	54.4	6.45	1	2.1	99	Z	107	13.8	10.7	18.66	54.0
W47285		1	M	2009	1.89866	0.82	59.5	6.97	0	2.6	90	Z	110	13.1	10.2	17.16	58.5
W47285	2	2	M	2010	2.23247	0.84	61.0	7.33	0	2.4	94	0	109	13.5	10.5	17.97	56.5
W47099	1	1	M	2009	3.21425	0.89	61.0	8.10	1	2.7	90	Z	114	13.6	11.0	18.33	59.0
W47099		2	M	2010	2.83257	0.89	61.3	8.17	0	2.6	83	Z	112	13.8	10.6	17.61	58.5
W47284		1	M	2009	1.88403	0.83	57.7	6.77	1	2.4	95	0	111	13.5	10.5	18.39	52.5
W47284	2	2	M	2010	1.90409	0.82	59.0	7.14	0	2.5	94	Z	112	13.6	10.2	17.55	58.5
T433596	5	5	M	2010	1.72219	0.82	58.5	6.22	1	2.9	86	0	108	12.7	10.3	19.35	54.0
T435271	5	5	M	2010	1.90570	0.80	59.8	7.34	1	2.4	96	Z	112	14.5	10.8	18.59	58.0
